# Peatland Mid-Infrared Database

**DOI:** 10.1038/s41597-026-06986-x

**Published:** 2026-04-06

**Authors:** Henning Teickner, Svenja Agethen, Sina Berger, Rieke Inga Boelsen, Werner Borken, Luca Bragazza, Tanja Broder, Florentino B. De La Cruz, Andrei-Cosmin Diaconu, Nancy B. Dise, Simon Drollinger, Cristian Estop-Aragonés, Mariusz Gałka, Magalí Martí, Stephan Glatzel, Jessica Groß, Lorna Harris, Liam Heffernan, Suzanne B. Hodgkins, Annkathrin Hömberg-Grandjean, Helga Hoppe, Till Kleinebecker, Wolfgang Knierzinger, Haojie Liu, Paul Mathijssen, Christopher Mollmann, Wiebke Schuster, Lisa Närtker, David Olefeldt, Verónica Pancotto, Nicolas Pelletier, Hendrik Reuter, Bjorn Robroek, Bo H. Svensson, Julie Talbot, Lauren Thompson, Fred Worrall, Zhi-Guo Yu, Klaus-Holger Knorr

**Affiliations:** 1 Ecohydrology & Biogeochemistry Group, Institute of Landscape Ecology, University of Münster, Münster, Germany; 2 Spatiotemporal Modelling Lab, Institute for Geoinformatics, University of Münster, Münster, Germany; 3 Pädagogische Hochschule St.Gallen (PHSG), Notkerstrasse 27, 9000 St.Gallen, Switzerland; 4 Albert-Ludwigs-Universität Freiburg, Freiburg, Germany; 5 Soil Ecology, University of Bayreuth, Dr.-Hans-Frisch-Str. 1-3, 95448 Bayreuth, Germany; 6 Agroscope, Field-Crop Systems and Plant Nutrition, Nyon, Switzerland; 7 University of North Florida: Jacksonville, Florida, US; 8 Department of Geology, Babeş-Bolyai University, Kogalniceanu, 1, 400084 Cluj-Napoca, Romania; 9 UK Centre for Ecology & Hydrology Bush Estate, Penicuik, Edinburgh, EH26 0QB United Kingdom; 10 Bioclimatology, University of Göttingen, Göttingen, Germany; 11 University of Lodz, Faculty of Biology and Environmental Protection, Department of Biogeography, Paleoecology and Nature Conservation, Banacha 1/3, 90-237 Łodz, Poland; 12 Department of Biomedical and Clinical Sciences, Linköping University, Linköping, Sweden; 13 Department of Thematic Studies, Environmental Change, Linköping University, 58183 Linköping, Sweden; 14 Department of Geography and Regional Research, Geoecology, Faculty of Earth Sciences, Geography and Astronomy, University of Vienna, Vienna, Austria; 15 Department of Renewable Resources, University of Alberta, Edmonton, AB T6G 2G7 Canada; 16 Department of Earth Sciences, Earth and Climate Cluster, Vrije Universiteit Amsterdam, Amsterdam, The Netherlands; 17 Department of Microbiology, The Ohio State University, Columbus, OH 43210 USA; 18 Institute for Landscape Ecology and Resources Management (ILR), Research Centre for BioSystems, Land Use and Nutrition (iFZ), Justus Liebig University Giessen, Heinrich-Buff-Ring 26, 35392 Giessen, Germany; 19 Centre for International Development and Environmental Research (ZEU), Justus Liebig University Giessen, Senckenbergstrasse 3, 35390 Giessen, Germany; 20 Federal Agency for Water Management, Institute for Land and Water Management Research, Petzenkirchen, 3252 Austria; 21 Faculty of Agricultural and Environmental Sciences, University of Rostock, Justus-von-Liebig-Weg 6, 18059 Rostock, Germany; 22 Climate Resilience, Wageningen Environmental Research, Wageningen University & Research, PO Box 47, NL-6700 Wageningen, AA Netherlands; 23 Stiftung Naturschutz Schleswig-Holstein, Eschenbrook 4, 24113 Molfsee, Germany; 24 Centro Austral de Investigaciones Científicas (CADIC), Consejo Nacional de Investigaciones Científicas y Técnicas (CONICET), Ushuaia, Tierra del Fuego Argentina; 25 Instituto de Ciencias Polares, Ambiente y Recursos Naturales (ICPA), Universidad Nacional de Tierra del Fuego (UNTDF), Ushuaia, Tierra del Fuego Argentina; 26 Département de géographie, Université de Montréal, Montréal, Canada; 27 Department of Ecohydrology and Biogeochemistry, Leibniz Institute of Freshwater Ecology and Inland Fisheries, Berlin, Germany; 28 Department of Ecology, Radboud Institute for Biological and Environmental Sciences, Faculty of Science, Radboud University Nijmegen, 6525 Nijmegen, AJ The Netherlands; 29 School of Biological Sciences, Faculty of Environmental and Life Sciences, University of Southampton, Southampton, SO17 1BJ UK; 30 Département de Géographie, Université de Montréal, Campus MIL, 1375 Avenue Thérèse Lavoie-Roux, Montréal, Québec H2V 0B3 Canada; 31 University of Alberta, Department of Renewable Resources, South Academic Building 348D, Edmonton, AB T6G 2G7 Canada; 32 Hatfield Consultants, 1228 Kensington Rd NW Unit 305, Calgary, AB T2N 3P7 Canada; 33 Department of Earth Sciences, University of Durham, Durham, DH1 3LE UK; 34 Ecohydrology Research Group, Dept. Hydrology & Water Resources, Nanjing University of Information Science and Technology, Ningliu Road. 219, 210000 Nanjing, China

**Keywords:** Carbon cycle, Element cycles, Environmental chemistry, Biogeochemistry, Freshwater ecology

## Abstract

Systematic collections of peat mid-infrared spectra and other peat properties are scarce, but useful to understand peat chemistry and develop spectral prediction models. The Peatland Mid-Infrared Database (‘pmird’) stores 3877 mid-infrared spectra of peat, peat-forming vegetation, and dissolved organic matter, together with measurements of other peat properties that were collated from previous studies. Most of the peat samples are from northern bogs, whereas southern or tropical peat and fen peat is underrepresented. The data are supplemented with metadata on sample origin, sample processing, measurements, and quality indicators on whether spectra are baseline corrected or not and on the relative contribution of water vapor, carbon dioxide, and noise to the spectra. The ‘pmird’ database can be used to analyze peat properties, develop and test spectral prediction models, and develop data and metadata standards.

## Background & Summary

Compared to many other soils, peat has a high carbon density^1^, and peatlands therefore store more than 500 Gt of carbon^2,3^, despite covering only 3% of the land surface^4^. Moreover, because a large part of peat is preserved because of high water table levels that slow down decomposition of the peat organic matter, peatlands can emit comparatively large amounts of carbon when water table levels decrease^5^, for example due to land use or climate change^6^. As for other ecosystems, characterizing peatland states and processes and developing process models requires measurements of many peat properties. In addition, a better understanding of processes requires information on peat molecular structures, for example decomposition processes^7^, redox reactions^8,9^, or metal accumulation^10^.

Mid-infrared spectra (MIRS) are useful for peatland studies because they allow quantification of the relative abundances of many molecular structures. MIRS have been used to estimate the degree of decomposition^11–13^, the amount of organic matter fractions^14^, and — with spectral prediction models — various peat properties, such as element contents, sugar fractions, or pH^14–16^. A database of peat MIRS would facilitate the synthesis of findings across individual studies and the development of robust spectral prediction models, thereby reducing the time and resources required to measure peat properties relevant to characterize peatland states and processes.

Peat samples are underrepresented even in the largest open access soil spectral libraries^16–22^. In addition, many libraries currently do not provide many of the variables relevant for peatland studies. Existing open databases focusing on peatlands^1,23,24^ do not contain spectral data. The Peatland Mid-Infrared Database (‘pmird’ database)^25^ addresses this data gap: it contains 3877 mid-infrared spectra of peat, peat-forming vegetation, and dissolved organic matter from peat porewater or peat-covered catchments, as well as data on various peat chemical and physical properties (Table 1).

**Table 1:** Summary of the datasets included into the pmird database.

ID	No. Samples	No. MIRS	Properties of peat, vegetation, DOM, or other samples	MIRS mode	References
1	397	397	Klason_lignin_content, holocellulose_content, age_14C, trace elements	ATR-FTIR	^27,28^
2	138	45	loss_on_ignition, age_14C, lab_code_14C, trace elements	Absorbance-FTIR	^29,30^
3	469	289	N, C, S, P, d13C, d15N, bulk_density, age_14C, lab_code_14C, trace elements	Absorbance-FTIR	^31–33^
4	216	212	N, C, d13C, d15N, mass, volume, bulk_density, loss_on_ignition, pH, trace elements	Absorbance-FTIR	^34,35^
5	78	78	N, C, S, P, d13C, d15N, bulk_density, pH, age_14C, lab_code_14C, porosity, trace elements	Absorbance-FTIR	^36^
6	36	36	N, C, S, P, pH, water_content, trace elements	Absorbance-FTIR	^37^
7	785	227	N, C, S, P, bulk_density, loss_on_ignition, age_14C, lab_code_14C, activity_210Pb, mass_210Pb, CaCO3, water_content, macrofossils, trace elements	Absorbance-FTIR	^38–40^
8	146	96	N, C, d13C, d15N, mass, volume, bulk_density, pH, water_content, trace elements	Absorbance-FTIR	
9	59	59	N, C, O, H, S, P, d13C, d15N, electron_accepting_capacity, electron_donating_capacity, Fe2, Fe3, trace elements	—	^41,42^
10	791	108	N, C, bulk_density, loss_on_ignition, age_14C, macrofossils, trace elements	—	^43,44^
11	79	79	N, C, d13C, d15N, trace elements	Absorbance-FTIR	^45^
12	557	191	N, C, S, P, d13C, d15N, mass, volume, bulk_density, age_14C, water_content, macrofossils, trace elements	Absorbance-FTIR	^46^
13	320	309	N, C, S, bulk_density, activity_210Pb, mass_210Pb, background_activity_reached_210Pb, activity_137Cs, year_137Cs, activity_226Ra, trace elements	—	
14	98	97	N, C, S, P, d13C, d15N, Fe2, Fe3, trace elements	Absorbance-FTIR	
15	102	90	N, C, S, P, d13C, d15N, mass, volume, bulk_density, age_14C, trace elements	Absorbance-FTIR	
16	298	96	N, C, S, P, mass, volume, bulk_density, activity_210Pb, mass_210Pb, activity_137Cs, year_137Cs, activity_226Ra, water_content, trace elements	ATR-FTIR, Absorbance-FTIR	^47,48^
17	114	114	trace elements	Absorbance-FTIR	^49^
18	1102	123	N, C, S, P, d13C, d15N, bulk_density, loss_on_ignition, age_14C, lab_code_14C, macrofossils, trace elements	Absorbance-FTIR	^50^
19	523	82	N, C, S, P, d13C, d15N, age_14C, lab_code_14C, macrofossils, trace elements	Absorbance-FTIR	^51^
20	634	124	N, C, S, P, d13C, d15N, bulk_density, loss_on_ignition, age_14C, lab_code_14C, macrofossils, trace elements	Absorbance-FTIR	^52^
21	138	48	bulk_density, trace elements	Absorbance-FTIR	^53,54^
22	106	85	N, C, O, H, S, trace elements	Absorbance-FTIR	^55,56^
23	1955	380	N, C, d13C, d15N, bulk_density, loss_on_ignition, age_14C, lab_code_14C, macrofossils, trace elements	Absorbance-FTIR	
24	54	54	N, C, trace elements	Absorbance-FTIR	^57–59^
25	380	0	bulk_density, loss_on_ignition, hydraulic_conductivity, porosity, macroporosity, trace elements	—	^26^
26	1641	446	N, C, S, bulk_density, activity_210Pb, mass_210Pb, trace elements	Absorbance-FTIR	^60,61^

“ID” is a unique identifier for each dataset in pmird. “No. MIRS” is the number of samples for which a MIRS is available. “Properties of peat, vegetation, DOM, or other samples” are the sample properties which were measured at least for one sample in the dataset. “MIRS mode” describes the MIRS measurement mode (ATR-FTIR: Attenuated total reflectance-Fourier transformed infrared, Absorbance-FTIR: Absorbance-Fourier transformed infrared, empty cells (“—”) mean that no information is available from the original dataset or that “MIRS mode” is not applicable because the dataset contains no MIRS). “Reference” are original references for the dataset. For data not previously described in a publication, “Reference” is empty.

The ‘pmird’ database is a legacy database that combines data from past studies, many of which are not yet published. The database contains samples from 26 studies worldwide. Most MIRS are transmission Fourier-transform MIRS, but there are also attenuated total reflectance Fourier-transform infrared spectra (ATR-FTIR). In addition to MIRS, ‘pmird’ contains heterogeneous data on peat physical (bulk densities, radiocarbon ages, ^210^Pb, ^226^Ra, ^137^Cs activities, volumetric water content), chemical (main and trace elemental contents, pH, loss on ignition, electron accepting and donating capacities), and paleoecological (plant macrofossils, testate amoebae) variables, depending on availability for each study.

Due to the legacy nature of the datasets, (meta)data completeness, quality, and validation vary between studies. The ‘pmird’ database provides as detailed metadata as possible to allow judging which samples and measurements meet specific quality requirements. In addition metadata that summarize the quality of the spectra are provided. Most of the peat samples are from northern bogs, whereas southern and tropical peat and fen peat is underrepresented.

We highlight two applications of the ‘pmird’ database: first, the database may be used to develop spectral prediction models that predict peat properties from MIRS, for example carbon contents. Second, missing measurements for peat properties in the ‘pmird’ database can be predicted from already available spectral prediction models to fill data gaps. Since the ‘pmird’ database contains many samples with spectra, this may allow the creation of a more comprehensive collection of peat samples with estimates for many more peat properties than currently available.

The ‘pmird’ database is a first attempt to make peat-related MIRS more accessible to researchers, to support the development of community standards for spectral data, and to facilitate research on peatland biogeochemistry. Contributions are welcome and can be proposed via https://github.com/henningte/pmird.

## Methods

The ‘pmird’ database was created by collecting data and metadata of completed and ongoing projects of the biogeochemistry and ecohydrology working group at the Institute for Landscape Ecology (University Münster), collaborating partners at various institutes, and open access data sources.

### Collection of suitable datasets

**Fig. 1:**
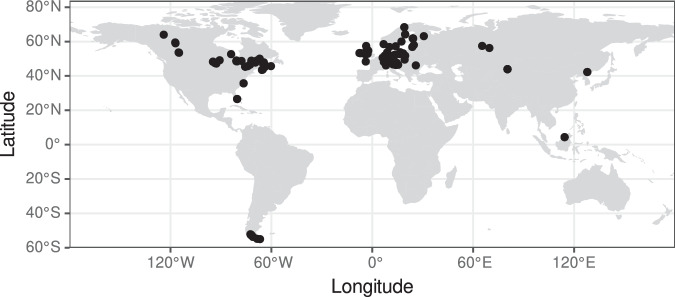
Map of all sampling locations for samples included in the ‘pmird’ database. Source: https://www.naturalearthdata.com.

First, a list of potentially available datasets was created. At this stage, the only criterion for inclusion on the list was the availability of MIRS of peat samples (except for data from Liu and Lennartz^26^, see below) or related samples (dissolved organic matter, peat-forming vegetation; data from Hodgkins *et al*.^27^ also contain paper and non-peatland vegetation samples that were used in peatland research) and that a data source was known to the first author. From this list, datasets were excluded when no permission for publication could be obtained. Datasets were included if they met the following conditions: either the data are published under an open access license compatible with the CC-BY 4.0 license, or the authors or responsible parties allowed publication of their data under the CC-BY 4.0 license. Data authors or responsible parties were contacted via email describing the project’s scope and aims, were asked for permission to use the data and to provide access to data and relevant metadata. Key characteristics of the datasets included in the ‘pmird’ database and references to original data sources are presented in Table 1 and an overview on the spatial distribution of samples is given in Fig. 1.

### Available data formats

For the remaining datasets, data were available as:

1. raw data outputs from measurement devices,
2. processed data outputs from measurement devices (e.g., baseline corrected MIRS, element contents predicted from wavelength-dispersive X-ray fluorescence analysis, etc.),
3. entries in template files specifically developed for the database,
4. custom files, mostly Excel spreadsheets and published manuscripts, created within the respective projects for which the data were originally collected.

Dataset contributions were preferred in the order from 1 to 4. Where possible, raw data were requested directly from data contributors or retrieved from device backups. If only available as PDF, values from data tables were extracted with the ‘tabulizer’ R package^62^. In a next step, these datasets were reorganized and included in the ‘pmird’ database.

### Database schema

**Fig. 2:**
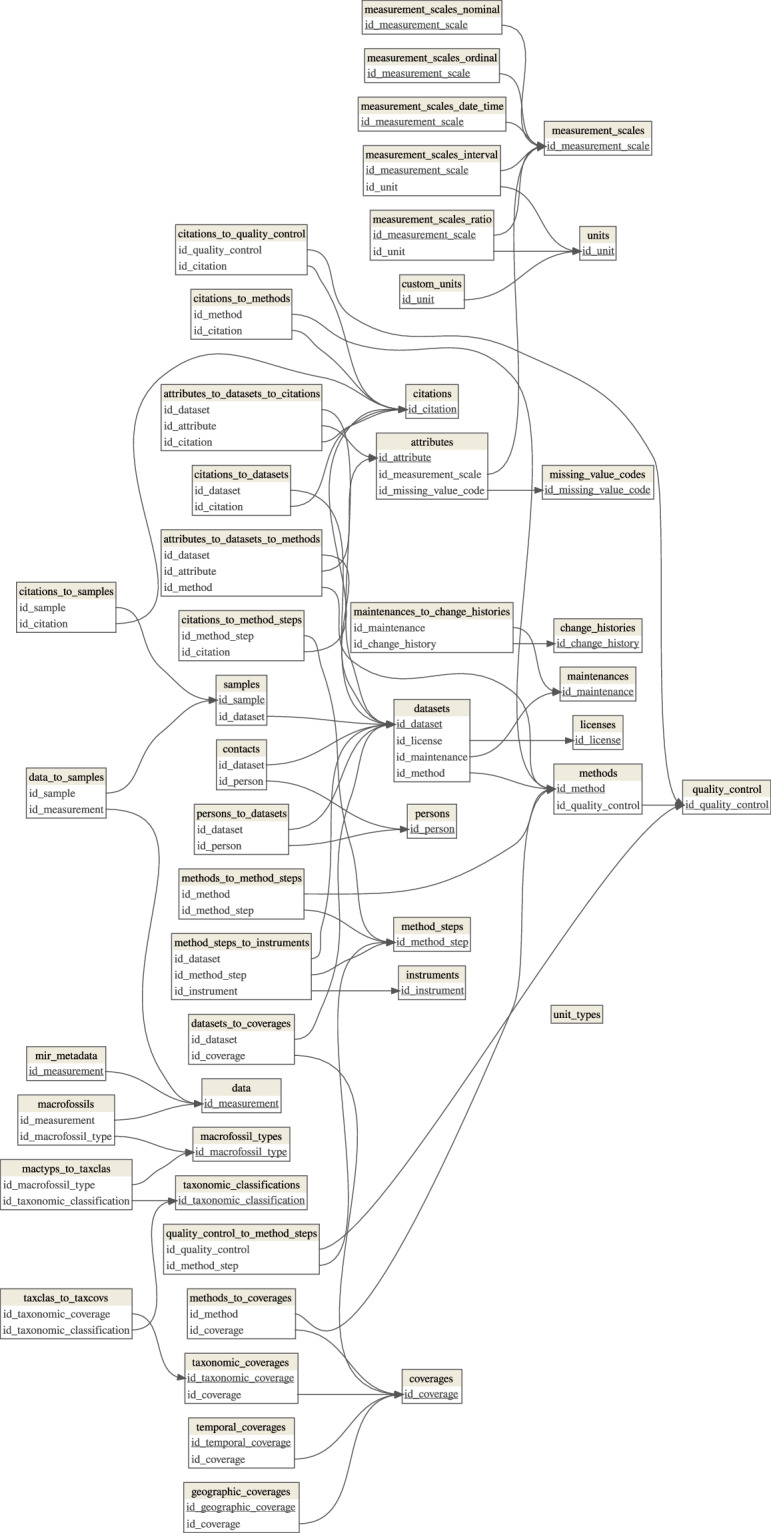
Database schema for ‘pmird’. Each table is represented as a box and contain unique identifiers for data entities (e.g. datasets, samples, or measurements) — primary keys (underlined) and foreign keys — which are listed in each box. Keys are used to link data entities between tables. These links are shown as curves pointing to the key in the parent table that is referenced.

The ‘pmird’ database was set up as a ‘MariaDB’ database. The database schema (Fig. 2) was designed to store data and metadata in accordance with classes and elements defined by the Ecological Metadata Language (EML)^63^ that were applicable and relevant to the data types and metadata available. General metadata that were considered relevant are geographic, temporal and taxonomic coverage, measurement instruments, and a description of individual methods and method steps^63^. In addition to the EML, discipline- and sample- or analytic-specific data reporting standards and recommendations were used to decide which data and metadata to include in the ‘pmird’ database:

1. peat coring and general peat geochemical and physical analysis^64–66^.
2. ^210^Pb, ^226^Ra, and ^137^Cs dating^67,68^.
3. radiocarbon dating^68^.
4. plant macrofossils and testate amoebae^69,70^.
5. MIRS: all metadata extractable with the R packages ‘hyperSpec’^71^ and ‘simplerspec’^72^ (as implemented in the R packages ‘ir’^73^ and ‘pmird’^74^) were extracted and additional quality variables were defined (see section Technical Validation).

A more detailed description of the database schema is given in section Data Records.

### Dataset import and preprocessing

The collected datasets were included into the ‘pmird’ database using functions from the ‘pmird’ package^74^. Where MIRS were available as raw data, they were imported including their metadata, using the R packages ‘ir’^73^, ‘hyperSpec’^71^, and ‘simplerspec’^72^. The metadata collected for MIRS are the metadata extracted with these packages. If metadata were additionally described in articles and reports using the data (for example the number of scans or measurement devices used), these were added. All spectra included in the database were included as received by the data contributors so that no additional preprocessing was applied to the MIRS, but if no raw spectra were available the MIRS may be already preprocessed to some extent.

Other preprocessing steps that were applied are:

1. The recalibration of C and N content data and *δ*^13^C and *δ*^15^N values using the R package ‘elco’^75^ — in the case where raw data were available. In C and N measurements coupled to isotope ratio mass spectrometry measurements, chromatogram peak areas and area ratios are used to estimate element contents and isotope values by calibrating measurements for standard materials with known C and N contents and *δ*^13^C and *δ*^15^N values. In peatland research and for the data included in the ‘pmird’ database, the calibration procedures are not well documented in most cases and may vary between datasets. For most datasets, the underlying raw data to reproduce the calibration were not available. Where raw data were available, we recalibrated the data with procedures from the ‘elco’ package to harmonize the calibration procedure. This calibration does not account for so-called blank effects^76^ and therefore the isotope values may be biased (depending on the C or N content and the mass of the sample). Correcting blank effects was not possible because no appropriate correction models could be constructed due to lack of sufficient standard measurements.
2. The recalibration of element contents analyzed by wavelength-dispersive X-ray fluorescence — in case pellet masses used during measurements differed from those used for calibration (also using ‘elco’). Measurement errors estimated from these recalibrations are stored alongside the corrected mean values.
3. Unit conversion conducted with the R packages ‘units’^77^ and ‘elco’.

During data import, some samples and data were excluded. These included: (1) samples for which no (approximate) sampling location and (approximate) sampling time were available, (2) corrupted MIRS (broken file format), (3) macrofossil counts where no exact sample volume was available, and (4) data which were not considered as usable in the original projects (e.g., due to measurement errors, device failures, etc.).

An exception was made for data from Liu and Lennartz^26^ that report a database of peat physical and hydraulic properties, albeit without MIRS, sampling locations nor dates. These data were included because they are not published yet, but can be useful to develop prediction models for peat hydraulic properties. None of the data from Liu and Lennartz^26^ correspond to other samples in the database.

In most cases, metadata were available only in the form of published manuscripts, including sampling locations, the description of methods, instruments, settings, and data preprocessing. From these data sources, as much detailed metadata as possible were extracted. In particular, any remarks available on data quality, validation, and processing were included either in the methods description or as comments for individual samples or measurements. In some cases, additional metadata was retrieved from data contributors.

## Data Records

The ‘pmird’ database^25^, including the externally stored MIRS data (see below) are made available on Zenodo. In addition, the ‘pmird’ R package^74^, an interface to the ‘pmird’ database (see section Usage Notes), is also available from Zenodo.

### Database schema

The schema of the ‘pmird’ database is shown in Fig. 2. The database consists of a set of individual tables visualized by boxes which are linked via keys (unique identifiers) listed within the boxes. These links are presented as curves between the tables. A description of the attributes of all tables is presented in Tab. S1.

The top-level table is datasets. It stores general information on a dataset, such as the dataset ID, title, year of publication, license, and reference publication. It also includes an identifier that links to the methods used to create the data.

#### Samples

To each dataset, a set of samples in the samples table is assigned via the variable id_dataset. The table samples stores metadata on individual samples collected during a project, such as where and when samples were collected, the sample type (e.g. peat or vegetation), taxonomic information, and the microform (e.g., hummocks, hollows, or lawns) from which the samples were collected. Special metadata or metadata difficult to standardize are stored in the comments_samples attribute. The table has a row for each sample indexed by id_sample.

#### Measurements

Attribute values derived from measurements (e.g., element contents, pH, bulk density) are stored in a separate table data, where each row represents a measurement and is indexed by id_measurement. This format allows storage of replicate measurements on the same sample as are common for some assays or sample collection protocols. The link between measurements and samples is provided via the table data_to_samples.

There are three exceptions to this setup: First, MIRS are not stored directly in data, but data only contains an attribute mirs_file that stores the relative path to the files that contain the MIRS within the data folder provided along with the database on Zenodo. Second, MIRS metadata are stored in a separate table mir_metadata linked to data via id_measurement. Third, plant macrofossil and testate amoebae data are stored in a separate table macrofossils linked to data via id_measurement to account for the high diversity in macrofossil attributes (e.g., different taxa, size classes, etc.).

#### Metadata

Additional metadata on coverage, methods, instruments, and involved persons are stored in separate tables. Each dataset has coverage information, including geographic coverage (a bounding box for the sampling locations and a description of the sampling locations; table geographic_coverages), temporal coverage (time point or range of the data collection; table temporal_coverages), and taxonomic coverage (taxa included in the dataset; table taxonomic_coverages). In cases where only the sampling year was known, the month and day were set to January 1 of that year. Such cases have a corresponding note in column comments_samples.

Each dataset has a detailed method description in several rows in the table methods. For each dataset, the first row contains a description of the sample collection and — if applicable, sampling design or experimental design. This row is linked to the table method_steps (via table methods_to_method_steps) where individual steps for all applied methods (if applicable) are described (e.g., drying, milling, MIRS measurements, measurements of elemental contents, plant macrofossil analysis, dating, etc.). An example of such a description is shown in Tab. S2.

In addition, table attributes_to_datasets_to_methods links individual methods for each dataset to the specific attributes they refer to (e.g., C content) to avoid ambiguity over which methods were used to measure a specific attribute.

Instruments used for data collection are listed in table instruments and linked to individual method steps in table method_steps via table method_steps_to_instruments. Finally, information on units for all attributes are stored in various tables linked by table measurement_scales.

## Technical Validation

All datasets were validated on two levels: a validation within the projects in which the data were originally created, and a validation prior inclusion in the database. Available information on the technical validation for each dataset is given within the methods and method_steps tables (Fig. 2).

### Validation within individual projects

The ‘pmird’ database combines heterogeneous datasets collected within different individual projects. For different attributes, the extent to which community standards for validation procedures exist is highly variable. For instance, peat dating typically has highly standardized validation procedures because measurements are performed by highly specialized laboratories with standardized protocols, whereas no single, widely adopted standard exists for example for bulk density measurements or for the measurement of MIRS. For these reasons, validation procedures, the extent to which these are reported, and data quality vary between datasets.

### Validation of datasets during import

As described in the section Collection of suitable datasets, a minimal quality check was whether sufficient metadata were available to provide basic information on the methods used (including instruments used, sample processing, sampling location and date). Due to the legacy nature of many datasets, an independent, full technical validation could not be performed, because in many cases raw data or information on data processing were not available.

Raw data (including standard measurements) were available for only a few datasets, and only for C and N contents and stable isotope signatures. In these cases, the calibration was checked and updated where necessary and possible and the corresponding estimated measurement errors were included in the database.

### Validation of mid-infrared spectra

We computed several quality indicators to check the quality of the MIRS: it was checked whether the imported spectra were baseline corrected and we estimated the relative contribution of water vapor or CO_2_ artifacts, and signal noise. These quality indicators can be used to filter MIRS in the ‘pmird’ database.

Below, we describe how the quality indicators were computed. All MIRS validation procedures make use of the following variables:

1. X_**1**_: The set of all MIRS interpolated with ir::ir_interpolate() with parameter dw set to 1.
2. A(X_1_): A vector that stores the sum of the intensity values of each spectrum in X_**1**_ after (1) clipping X_**1**_ to the range [699, 3999] cm^−1^, (2) linearly interpolating the region of the CO_2_ peaks ([2250, 2450] cm^−1^) to avoid corrupted baselines due to negative CO_2_ peaks, (3) baseline correction using ir::ir_bc_rubberband().

The database contains only two datasets with ATR spectra and only for one are detailed information available on ATR crystal properties and preprocessing, including ATR correction. These metadata are stored within the methods and method_steps tables. Future versions of the database should store this information in a formalized way to facilitate preprocessing of ATR spectra.

#### Definition of reference spectra

**Fig. 3:**
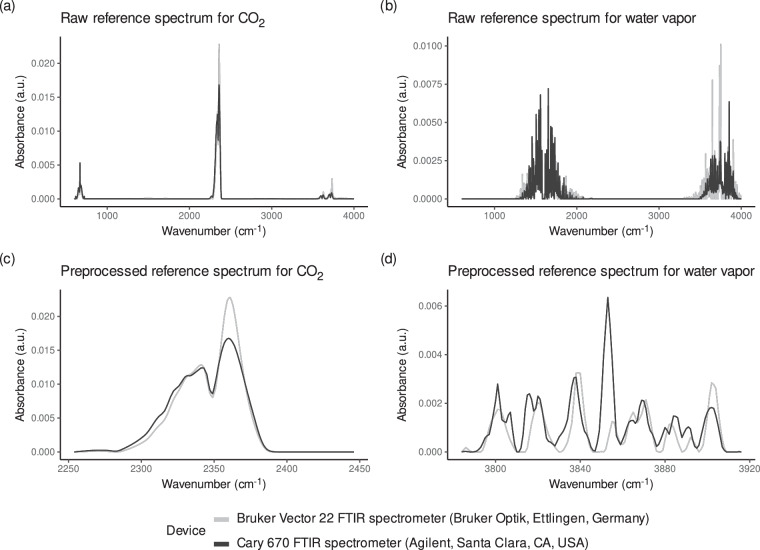
The raw and preprocessed reference spectra used to estimate the relative contribution of CO_2_ and water vapor to the spectra in the ‘pmird’ database. The preprocessed spectra (bottom row) correspond to ${{\rm{x}}}_{{{\rm{CO}}}_{2}}$ and x_wv_, respectively. The top row shows the same spectra before preprocessing. Colors indicate the measurement devices on which the spectra were recorded.

**Fig. 4:**
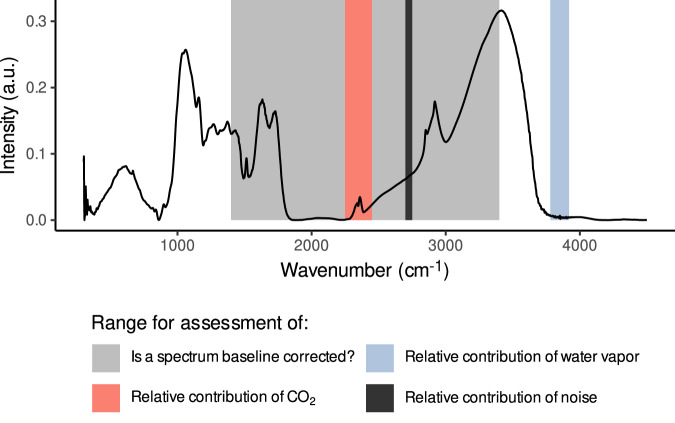
A sample spectrum from the ‘pmird’ database with the spectral ranges used during assessment of the quality attributes highlighted. For “Is a spectrum baseline corrected?”, the region of the spectra is clipped to [1400, 3400] cm^−1^. To assess the relative contribution of water vapor, the range [3780, 3920] cm^−1^ is considered. To assess the relative contribution of CO_2_, CO_2_ peaks in the range [2250, 2450] cm^−1^ are considered. To assess the relative contribution of noise, the range [2700, 2750] cm^−1^ is considered.

We quantified water vapor and CO_2_ artifacts using reference spectra of pure water vapor and CO_2_ (Fig. 3). The general procedure is to scale these reference spectra to a selected portion of the spectra which contains only water vapor or CO_2_ peaks (Fig. 4). The so computed scale factor is an estimate for the relative contribution of water vapor and CO_2_, respectively.

Ideally, the spectra of pure water vapor and CO_2_ gas would be measured on each used device on which the (peat) sample MIRS were measured^78^. However, no such data were available. When background scans were present, we derived equivalent data for CO_2_ from raw MIRS according to the following procedure:

1. Selection: Select two background spectra recorded on the same device and preferentially on the same day (with intensities recorded as transmission) which were recorded under different CO_2_ concentrations, but similar water vapor concentrations. The differences can be inferred from the relative magnitude of peaks which are known to be caused by water vapor and CO_2_, respectively^79,80^. Since we are only interested in an approximate estimation of the relative contribution of CO_2_, remaining small differences in water vapor concentrations were negligible (Fig. 3).
2. Calculation: Compute the spectrum for pure CO_2_ by dividing the intensity values of both spectra. This spectrum is converted to absorbance values.
3. Preprocessing: Perform additional preprocessing steps to make the spectra usable for the estimation and potential correction tasks: (1) interpolate the spectrum to integer wavenumber values increasing by 1 cm^−1^, (2) replace the regions 728 to 2230 cm^−1^ and  > 3800 cm^−1^ with straight lines (this was done to remove remaining noise and water vapor artifacts from regions known not to contain peaks caused by CO_2_, only in case such artifacts were visible in the resulting spectra^79,80^), and (3) baseline correct the spectra.

For water vapor, the procedure to obtain an approximately pure water vapor spectrum is the same as for CO_2_, with the following differences: In step 3 above, no regions of the water vapor spectrum are replaced by straight lines. Moreover, the following additional steps were performed (after step 3 above):

1. Atmospheric correction: Use the respective pure CO_2_ spectrum to perform an atmospheric correction as elsewhere suggested^81^. This was done because CO_2_ concentrations differed to some extent between the available background spectra. This correction was only partly successful, but sufficient to obtain an approximately pure water vapor spectrum for our purposes.
2. Remaining negative intensity values were removed by dividing the intensity values such that the background at 2000 cm^−1^ had an intensity of 1, then subtracting 1 from the intensity values, and finally setting all values  < 0 to 0. Remaining CO_2_ artifacts and noise were removed by replacing the regions 600 to 1200 cm^−1^ and 2200 to 3300 cm^−1^ with straight lines.

The resulting spectra (Fig. 3) have key characteristics of pure water vapor (https://webbook.nist.gov/cgi/inchi?ID=C7732185&Type=IR-SPEC&Index=0) and CO_2_ spectra (https://webbook.nist.gov/cgi/cbook.cgi?ID=C124389&Type=IR-SPEC&Index=1) as contained in the NIST/EPA Gas-Phase Infrared Database^79,80^.

Such reference spectra could not be computed for every device used to measure MIRS in the ‘pmird’ database due to missing raw data. For this reason, we used reference spectra computed from data from different devices as reference spectra for other devices. Reference spectra could only be computed for absorbance spectra, but not ATR spectra. As a consequence, water vapor and CO_2_ MIRS artifacts in ATR spectra are estimated using reference spectra measured in absorbance mode which introduces larger errors to our estimates in these cases. An overview of the assignment of reference spectra to devices is given in Table 2.

**Table 2:** Overview on the devices with which data to compute the reference spectra were measured ("Source device”) and for data from which devices these reference spectra are used to estimate the relative contribution of water vapor and CO_2_ artifacts (“Target device”).

Source device	Target device
Bruker Vector 22 FTIR spectrometer (Bruker Optik, Ettlingen, Germany)	Bruker Vector 22 FTIR spectrometer (Bruker Optik, Ettlingen, Germany)
	Shimadzu IRTracer-100 spectrophotometer, equipped with a DLaTGS (deuterated L-alaninedoped triglycine sulfate) detector
	PerkinElmer Spectrum 100 FTIR spectrometer
Cary 670 FTIR spectrometer (Agilent, Santa Clara, CA, USA)	Cary 660 FTIR spectrometer (Agilent, Santa Clara, CA, USA)
	Varian 670 FTIR spectrometer (Agilent, Palo Alto, USA)
	Cary 670 FTIR spectrometer (Agilent, Santa Clara, CA, USA)
	Cary 600 FTIR spectrometer (Agilent, Santa Clara, CA, USA)
	Varian 660 FTIR spectrometer (Agilent, Palo Alto, USA)

#### Identification of baseline corrected spectra

Different device settings, sample amounts, and internal scattering result in different baseline absorbances recorded during MIRS measurements^78^. Baseline correction is a heuristic procedure to subtract such differences in the baseline absorbance and thus is typically required to compare spectra of different samples^78^.

However, since baseline correction strongly depends on the specific spectra (e.g., the spectral range) and the occurrence of water and CO_2_ artifacts, and since different baseline procedures exist and more suitable algorithms may be developed in the future, it is preferable to publish unprocessed spectra.

Raw spectra were not available for all datasets in the ‘pmird’ database, as often only already baseline corrected data were available. Since these spectra can nevertheless be useful, they were not excluded. A further problem is that information on whether a spectrum had been already baseline corrected was unavailable, except when stated in published articles.

To identify spectra that were already baseline corrected, we used the fact that MIRS of organic matter typically have a baseline that decreases from the smallest MIR wavenumber ranges (~ 400 cm^−1^) to ~2300 cm^−1^. Thus, non-baseline corrected spectra have large baseline absorbances at lower wavenumber values, whereas already baseline corrected spectra have small baseline absorbances in this region.

We used the following procedure to detect spectra that were already baseline corrected:

1. Compute X_**2**_: Take X_**1**_, (1) interpolate linearly the region of the CO_2_ peaks (as described above), (2) perform the rubberband baseline correction as described above, but return the baseline instead of the baseline corrected spectra, (3) clip the baselines to the region [1400, 3400] cm^−1^ to get a uniform reference range (this is important to get homogeneous results after normalization in the following step, Fig. 4), and (4) divide the resulting baseline absorbances by A(X_1_).
2. Compute I_1400_(X_**2**_): Extract from X_**2**_ the normalized intensity at 1400 cm^−1^.
3. Define the logical vector is_baseline_corrected which is TRUE whenever I_1400_(X_**2**_) > t_bc_ and otherwise FALSE, where t_bc_ is set to 9 × 10^−5^. is_baseline_corrected is stored in the table mir_metadata in the database.

**Fig. 5:**
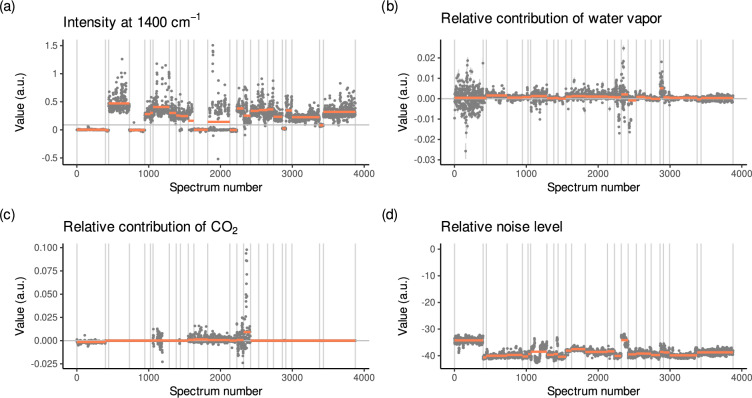
Data quality assessment for the spectra in the ‘pmird’ database. For each validation step described in section Validation of mid-infrared spectra, the values of the defined variable to assess the quality of the spectra are shown for each spectrum. Spectra are listed in the order of their appearance in the ‘pmird’ database along the horizontal axis. Vertical lines represent the first and last spectrum in a dataset. Red horizontal lines are the dataset averages. Error bars represent standard errors for estimated contributions of water vapor and CO_2_. (**a**) Intensity of the baseline of the spectra at 1400 cm^−1^ (I_1400_(X_**2**_)). This variable is used to identify baseline corrected spectra. The horizontal grey line denotes the threshold value (t_bc_ = 9 × 10^−5^) used to differentiate baseline corrected spectra (below the line) from non-baseline corrected spectra (above the line). For illustrative purposes, all values along the vertical axis are multiplied by 1000. (**b**) Relative contribution of water vapor to the spectra (c_wv_, mir_water_vapor_contribution_relative in table mir_metadata). (**c**) Relative contribution of CO_2_ to the spectra (${{\rm{c}}}_{{{\rm{CO}}}_{2}}$, mir_co2_contribution_relative in table mir_metadata). (**d**) Relative contribution of noise to the spectra (c_noise_, noise_level_relative in table mir_metadata). For illustrative purposes, all values along the vertical axis are log transformed.

The value of t_bc_ was defined based on visual inspection of Fig. 5(a) which shows I_1400_(X_**2**_) versus the MIRS measurement number in the ‘pmird’ database. Differences between datasets are clearly visible, as well as between baseline corrected spectra which have values near zero and non-baseline corrected spectra which have larger values than t_bc_. t_bc_ was not set to a smaller value because dataset 1 (containing ATR-FTIR spectra) contains baseline corrected spectra^27^, but has higher values for I_1400_(X_**2**_) due to noise.

#### Water vapor artifacts

Water vapor causes a range of artifact peaks in MIRS due to differences in the atmospheric water content during sample measurements in comparison to background measurements^78^. Water vapor artifacts can distort peaks from organic matter across broad regions of the MIRS^78^. High quality spectra have small water vapor peaks. To detect water vapor peaks, we focused on the range [3780, 3920] cm^−1^, where a series of water vapor peaks can be observed^78,79^, whilst organic matter typically causes no peaks in this range^82,83^ (Fig. 4).

We used the following procedure to estimate the relative contribution of water vapor to the MIRS:

1. Compute X_**3**_: Process X_**1**_ with pmird::pm_ir_extract_peak() with range set to [3780, 3920] cm^−1^, and peak_max to 3853 cm^−1^. This clips X_**1**_ to the defined water vapor region and baseline corrects the region conditional on whether water vapor artifacts are negative (less water vapor contribution in the sample spectrum in comparison to the background spectrum) or positive (more water vapor contribution in the sample spectrum in comparison to the background spectrum). Finally, normalize the intensities by division by A(X_1_).
2. Define x_wv_: A reference spectrum from X_**3**_ as described above in section Definition of reference spectra.
3. Define c_wv_: For each spectrum in X_**3**_, model the intensities with the intensities in x_wv_ using ordinary least squares regression, and extract the slope of the regression line (average and standard error).

c_wv_ is the relative contribution of water vapor to each spectrum in the database and SE(c_wv_) the standard error. For x_wv_, c_wv_ = 1. For a spectrum with no water vapor artifacts, c_wv_ ≈ 0. For spectra with negative water vapor artifacts, c_wv_ < 0, and for spectra with positive water vapor artifacts, c_wv_ > 0. An overview on the values of c_wv_ for all spectra is given in Fig. 5(b). c_wv_ is stored as mir_water_vapor_contribution_relative in the table mir_metadata in the database, and SE(c_wv_) as mir_water_vapor_contribution_relative_sd.

#### CO_2_ artifacts

CO_2_ causes a range of artifact peaks in MIRS due to differences in the atmospheric CO_2_ concentration during sample measurements in comparison to background measurements^79,81^. CO_2_ artifacts can distort peaks from organic matter particularly around ca. 600 to 750, 2250 to 2400, and 3500 to 3700 cm^−1^^79,80^. High quality spectra have small CO_2_ peaks. To detect CO_2_ peaks, we focused on the range [2250, 2450] cm^−1^, where a series of CO_2_ peaks can be observed^79,80^, whilst organic matter typically causes no peaks in this range^82,83^ (Fig. 4).

We used the following procedure to estimate the relative contribution of CO_2_ to the MIRS:

1. Compute X_**4**_: Process X_**1**_ with pmird::pm_ir_extract_peak() with range set to [2250, 2450] cm^−1^, and peak_max to 2362 cm^−1^. This clips X_**1**_ to the defined CO_2_ region and baseline corrects the region conditional on whether CO_2_ artifacts are negative (less CO_2_ contribution in the sample spectrum in comparison to the background spectrum) or positive (more CO_2_ contribution in the sample spectrum in comparison to the background spectrum). Finally, normalize the intensities by division by A(X_1_).
2. Define ${{\rm{x}}}_{{{\rm{CO}}}_{2}}$: A reference spectrum from X_**4**_ as described above in section Definition of reference spectra.
3. Define ${{\rm{c}}}_{{{\rm{CO}}}_{2}}$: For each spectrum in X_**4**_, model the intensities with the intensities in ${{\rm{x}}}_{{{\rm{CO}}}_{2}}$ using ordinary least squares regression, and extract the slope of the regression line (average and standard error).

${{\rm{c}}}_{{{\rm{CO}}}_{2}}$ is the relative contribution of CO_2_ to each spectrum in the database and ${\rm{SE}}({c}_{{{\rm{CO}}}_{2}})$ the standard error. For ${{\rm{x}}}_{{{\rm{CO}}}_{2}}$, ${{\rm{c}}}_{{{\rm{CO}}}_{2}}=1$. For a spectrum with no CO_2_ artifacts, ${{\rm{c}}}_{{{\rm{CO}}}_{2}}\approx 0$. For spectra with negative CO_2_ artifacts, ${{\rm{c}}}_{{{\rm{CO}}}_{2}} < 0$, and for spectra with positive CO_2_ artifacts, ${{\rm{c}}}_{{{\rm{CO}}}_{2}} > 0$. An overview on the values of ${{\rm{c}}}_{{{\rm{CO}}}_{2}}$ for all spectra in the database is given in Fig. 5(c). ${{\rm{c}}}_{{{\rm{CO}}}_{2}}$ is stored as mir_co2_contribution_relative in the table mir_metadata in the database, and ${\rm{SE}}({c}_{{{\rm{CO}}}_{2}})$ as mir_co2_contribution_relative_sd.

#### Noise

MIRS intensities can have contributions from signal noise, e.g., in dependency of the number of scans averaged per spectrum, the sensor, and the MIR radiation source^78^. Noise can distort peaks and can cause differences in baseline correction, as well as the identification of water vapor and CO_2_ artifacts. We estimated the relative noise contribution as the variance of intensity values around the average intensity in a spectrum in a region without sharp peaks. For this, we focused on the range [2700, 2750] cm^−1^ (Fig. 4), which has no sharp peaks caused by organic matter and is less impacted by water vapor than other regions without sharp peaks in organic matter MIRS.

We used the following procedure to estimate the relative contribution of noise to the MIRS:

1. Define c_noise_: The variance of intensity values in X_**1**_ after (1) clipping X_**1**_ to the range [699, 3999] cm^−1^, (2) interpolating linearly the region of the CO_2_ peaks ([2250, 2450] cm^−1^) — to avoid corrupted baselines due to negative CO_2_ peaks —, (3) baseline correction using ir::ir_bc_rubberband(), (4) Savitzky-Golay baseline correction to estimate the average intensity of a spectrum, (5) Normalization of intensity values by dividing them by their sum, (6) clipping to the noise range ([2700, 2750] cm^−1^), and (7) computation of the variance of the intensity values.

If c_noise_ ≈ 0, no noise is detected in the spectrum and the larger c_noise_ is, the larger is the relative contribution of noise. An overview on the values of c_noise_ for all spectra is given in Fig. 5(d). c_noise_ is stored as noise_level_relative in the table mir_metadata in the database.

## Usage Notes

The ‘pmird’ database can be downloaded from Zenodo^25^. The downloaded data contain a database backup (pmird-backup-2025-09-10.sql) and raw MIRS data files in the folder pmird_prepared_data. The database backup needs to be imported to a ‘MariaDB’ server, the folder pmird_prepared_data can be stored at any location. In a linux terminal, the downloaded database backup can be imported like so:


mysql -u<user> -p pmird < pmird-backup-2025-09-10.sql


Here, <user> is the user for the ‘MariaDB’ server. More information on ‘MariaDB’ can be found here: https://mariadb.com/.

Data can be accessed for example via ‘MariaDB’, or via R^84^ with the ‘RMariaDB’ package^85^. An R package that provides functions to access and manipulate the database, ‘pmird’^74^, has also been developed. The following use cases illustrate how to access the database with the ‘pmird’ package and what additional packages may be useful to analyze data exported from the database, in particular the spectra.

### Database access via the ‘pmird’ R package

The ‘pmird’ R package can be downloaded and installed from GitHub using the remotes package^86^. Other packages needed for this tutorial are also installed:

# installationremotes::install_github(“henningte/pmird”)# installation of other packagesinstall.packages(“magrittr”)install.packages(“RMariaDB”)remotes::install_github(“henningte/ir”)remotes::install_github(“henningte/irpeat”)# load needed packages for this tutoriallibrary(pmird)library(ir)library(irpeat)

Once the database is set up and runs in a ‘MariaDB’ instance (see previous subsection), it can be accessed from within R, using the ‘RMariaDB’ package^85^:

# connect to databasecon <-RMariaDB::dbConnect(drv = RMariaDB::MariaDB(),dbname = “pmird”,default.file = “~/my.cnf”,groups = “rs-dbi”)

Here, my.cnf is a text file that stores user and password information for the database server.

From here on, the ‘pmird’ R package can be used to access the database. The ‘pmird’ R package makes use of the R package ‘dm’^87^ to access and manipulate the database contents. pmird::pm_get_dm() creates a dm object which stores the database structure.

# create the dm objectdm_pmird <- pmird::pm_get_dm(con, learn_keys = TRUE)

The option learn_keys = TRUE means that information on the primary and foreign key is added to the dm object. A dm object is a representation of the entire database and allows comfortable manipulation of the database from within R (e.g., addition of new rows to tables, addition of new tables, data queries)^87^.

### Use case: obtaining general information on the datasets contained in the ‘pmird’ database

General information on the datasets contained in the ‘pmird’ database is stored in table datasets. The ‘pmird’ R package provides a function to obtain this table from the dm object (pmird::pm_get_table(.table_name = "datasets)).

# extract the datasets tablepmird_datasets <-dm_pmird |>pmird::pm_get_table(.table_name = “datasets”)

This table can, for example, be used to select studies to extract data from the ‘pmird’ database.

### Use case: extracting data for a specific dataset from the ‘pmird’ database

Assume you are interested in viewing all measured data for one specific dataset, e.g. the dataset with id_dataset == 8 in pmird_datasets. Using the ‘dm’ package and the dm object representing the ‘pmird’ database (dm_pmird), these data can be obtained as follows:

# get data for the dataset with ID 8d8 <-dm_pmird |>dm::dm_zoom_to(datasets) |>dm::filter(id_dataset == 8) |>dm::left_join(samples, by = “id_dataset”) |>dm::left_join(data_to_samples, by = “id_sample”) |>dm::left_join(data, by = “id_measurement”) |>dm::left_join(mir_metadata, by = “id_measurement”) |>dm::left_join(macrofossils, by = “id_measurement”) |>dm::pull_tbl() |>tibble::as_tibble()

The resulting data frame (d8) contains information on all samples and measurements for the dataset with id_dataset == 8. d8 does not yet contain any spectra, but stores only information on the respective file paths to the downloaded spectra files within the downloaded folder pmird_prepared_data.

To load the spectra, you can use the function pmird::pmird_load_spectra(). In addition, you have to specify via argument directory in which folder pmird_prepared_data is stored (on the computer used for this tutorial, this was "data/derived_data/"):

# load the spectrad8 <- pmird::pm_load_spectra(d8, directory = “data/derived_data/”)

pm_load_spectra() is a wrapper function around functions from the package ‘ir’^73^ which in turn are wrappers around read.spc()^71^ and read.csv(). pm_load_spectra() therefore can load spectra both saved as spc and csv files. pm_load_spectra() also converts d8 into an object of class ir from the ‘ir’ package. ‘ir’ provides functions for spectral preprocessing and manipulation^73^ and is compatible with the ‘irpeat’ package which provides functions to analyze peat MIRS and spectral prediction models to predict peat properties from MIRS^88^.

### Use case: spectral preprocessing workflow

Here, an example workflow to preprocess the spectra in d8 with the R package ‘ir’ is shown. The workflow assumes high quality MIRS and therefore does not correct noise, water vapor or CO_2_ artifacts. The workflow has the following steps:

1. Linear interpolation (When specifying wavenumbers, for example during clipping, ‘ir’ warns about any numeric deviations. Linear interpolation avoids these warnings).
2. Clipping to the wavenumber range of interest.
3. Baseline correction using a convex hull^71^.
4. Normalization by dividing all intensity values by the sum of all intensity values.

Which map to the following code:

# define the clipping rangeclip_range <-data.frame(start = 650,end = 3990,stringsAsFactors = FALSE)# typical preprocessing workflowd8_preprocessed <-d8 |>ir::ir_interpolate(start = NULL, dw = 1) |>     # linear interpolationir::ir_clip(range = clip_range) |>         # clippingir::ir_bc (                                       # baseline correctionmethod = “rubberband”,do_impute = TRUE) |>ir::ir_normalize(method = “area”)               # normalization

**Fig. 6:**
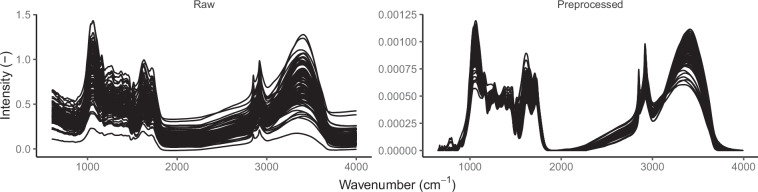
Comparison of the spectra for the dataset d8 extracted from the ‘pmird’ database before and after applying the preprocessing workflow described in section Use case: spectral preprocessing workflow.

A comparison of the loaded spectra before and after preprocessing is shown in Fig. 6.

### Use case: computation of humification indices and spectral prediction models

Here, computation of humification indices^11,12,45^ with the ‘irpeat’ package is shown:

# compute a humification indexd8_preprocessed <-d8_preprocessed |>irpeat::irp_hi(x1 = 1630, x2 = 1090)# show some valueshead(d8_preprocessed$hi_1630_1090, 3)## [1] 0.4653120 0.4507052 0.8271826

The ‘irpeat’ package contains, for example, a prediction model for the electron accepting capacity^42^ that can be applied to raw MIRS (for further details, please see the documentation of the ‘irpeat’ package):

d8_eac <-d8 |>dplyr::filter(! ir::ir_identify_empty_spectra(d8)) |>irpeat::irp_eac_1(do_summary = TRUE)# show some valueshead(d8_eac$eac, 3)## Units: [umol/g]## Errors: 172.0593 168.4222 191.2435##         1        2        3## 611.5772 536.4737 805.5097

### Use case: Handling units and measurement errors

The R package ‘quantities’^77,89^ can be used to add units and measurement errors to measured variables from the ‘pmird’ database. The ‘pmird’ R package allows batch unit and measurement error assignment:

# add information on units and errors with the ‘quantities’ packaged8 <-d8 |>pmird::pm_add_quantities()# show some valueshead(d8$N, 3)## Units: [g/g]## Errors: 0.0000996441 0.0001036096 0.0001168346## [1] 0.014054321 0.007934011 0.021408554

### Use case: Generating data citations

Whoever uses data from the ‘pmird’ database should cite, in addition to the database, the original data sources for the used datasets. To make this straightforward, the ‘pmird’ R package contains the function pm_get_citations() to generate such a citation list for any extracted data subset:

# collect all citations for `d8`:d8_citations <-pm_get_citations(con = con,x = d8$id_measurement,file = “d8_citations.bib”)## Loading required namespace: bib2df# close connection to databaseRMariaDB::dbDisconnect(con)

The function takes column id_measurement of the extracted data and collects citations for all relevant data sources from the database. The results are exported to a bibtex file which is defined via argument file. This file can be imported to literature reference software. In this case, since the data have not been previously published, the created bibtex file is empty. RMariaDB::dbDisconnect(con) closes the connection to the database, as this is the final use case presented here.

### Future contributions

Besides the addition of new datasets, there are several ways by which the database can and should be improved. While our effort was to collect available MIRS and related data, future developments should focus on harmonizing and standardizing measurement procedures. This is necessary because ring trials have repeatedly shown large variations in measurements on the same standard materials (and partly even with the same standard operating procedures)^90,91^. These efforts require a standardization of sample preprocessing and analytic methods, as for example suggested by GLOSOLAN and IEEE SA P4005^92,93^ (that would have to be supplemented to address peat-specific methods^94^). This may happen in different ways, for example by integrating spectral data into open spectral databases currently focusing on mineral soils that try to follow and contribute to the development of data and metadata standards for soil spectroscopy^91,95^ and by ring trials on peat standard materials.

Currently, the development of standards for spectroscopy is still under development and additionally complicated by differences in spectroscopic methods^91^. Suggesting specific reporting guidelines is outside the scope of this data descriptor. We suggest that minimal information to be provided on MIRS are the device model, beamsplitter and detector type, material of ATR diamonds, angle of incidence, the number of scans averaged per spectrum, spectral resolution, whether or not measurement cells were purged with synthetic air before measurements (and how long), the material used for background correction, and the background spectra itself. New data should be provided as raw data, including background spectra, in a data format that can be decoded with free and open source software and that ideally stores comprehensive metadata.

Future data submissions and other improvements of the database may be discussed on the GitHub repository of the ‘pmird’ package (https://github.com/henningte/pmird) or via email to the corresponding author. Future data submissions should follow the table and attribute format of the database and will be processed depending on free capacities. Since the ‘pmird’ database comes with open source code and open-data licenses, anybody interested in contributing can work with and on the database.

## Supplementary information

Supporting information to: Peatland Mid-Infrared Database

## Data Availability

The Peatland Mid-Infrared Database is available from Zenodo (10.5281/zenodo.17092587)^25^.

## Code availability

All code used to create the ‘pmird’ database is available within the repository of the ‘pmird’ database^25^. Detailed information about how these scripts work together can be found in the README file in the repository. The underlying data cannot be made available because some of them contain personal data. Therefore, the database cannot be reproduced from scratch. The ‘pmird’ R package is available from Zenodo^74^. Code to reproduce this manuscript is available from GitHub^96^.
