# Supplementary material for: Peatland Mid-Infrared Database
Source: Sci Data. 2026 Apr 6;13:538. doi: 10.1038/s41597-026-06986-x (PMC13057142; doi:10.1038/s41597-026-06986-x)
Supplement: Supplementary file 1 — Supporting information to: Peatland Mid-Infrared Database [file 41597_2026_6986_MOESM1_ESM.pdf]

Supporting information to: Peatland Mid-Infrared Database

Contents

Attributes in the ‘pmird’ database **3**

Example of the method description for dataset 8 **23**

Henning Teickner[1,2,*]
Svenja Agethen[1]
Sina Berger[3]
Rieke Inga Boelsen[4]
Werner Borken[5]
Luca Bragazza[6]
Tanja Broder[1]
Florentino B. De La Cruz[7]
Andrei-Cosmin Diaconu[8]
Nancy B. Dise[9]
Simon Drollinger[10]
Cristian Estop-Aragonés[1]
Mariusz Gałka[11]
Magalí Martí[12,13]
Stephan Glatzel[14]
Jessica Groß[1]
Lorna Harris[15]
Liam Heffernan[16]
Suzanne B. Hodgkins[17]
Annkathrin Hömberg-Grandjean[1]
Helga Hoppe[1]
Till Kleinebecker[18,19]
Wolfgang Knierzinger[20]
Haojie Liu[21]
Paul J.H. Mathijssen[1,22]
Christopher Mollmann[1]
Wiebke Schuster[23]
Lisa Närtker[1]
David Olefeldt[15]
Verónica Pancotto[24,25]

Nicolas Pelletier[26]
Hendrik Reuter[27]
Bjorn Robroek[28,29]
Bo H. Svensson[13]
Julie Talbot[30]
Lauren Thompson[31,32]
Fred Worrall[33]
Zhi-Guo Yu[34]
Klaus-Holger Knorr[1]

[1] Ecohydrology & Biogeochemistry Group, Institute of Landscape Ecology, University of Münster, Germany
[2] Spatiotemporal Modelling Lab, Institute for Geoinformatics, University of Münster, Germany
[3] Pädagogische Hochschule St.Gallen (PHSG), Notkerstrasse 27, 9000 St.Gallen, Switzerland
[4] Albert-Ludwigs-Universität Freiburg, Germany
[5] Soil Ecology, University of Bayreuth, Dr.-Hans-Frisch-Str. 1-3, 95448 Bayreuth, Germany
[6] Agroscope, Field-Crop Systems and Plant Nutrition, Nyon, Switzerland
[7] University of North Florida: Jacksonville, Florida, US
[8] Department of Geology, Babeş-Bolyai University, Kogalniceanu, 1, 400084, Cluj-Napoca, Romania
[9] UK Centre for Ecology & Hydrology Bush Estate, Penicuik, Edinburgh EH26 0QB, United Kingdom
[10] Bioclimatology, University of Göttingen, Göttingen, Germany
[11] University of Lodz, Faculty of Biology and Environmental Protection, Department of Biogeography, Paleoecology and Nature Conservation, Banacha 1/3, 90-237 Łodz, Poland
[12] Department of Biomedical and Clinical Sciences, Linköping University, Linköping, Sweden
[13] Department of Thematic Studies, Environmental Change, Linköping University, 58183 Linköping, Sweden
[14] Department of Geography and Regional Research, Geoecology, Faculty of Earth Sciences, Geography and Astronomy, University of Vienna, Vienna, Austria
[15] Department of Renewable Resources, University of Alberta, Edmonton, AB, T6G 2G7, Canada
[16] Department of Earth Sciences, Earth and Climate Cluster, Vrije Universiteit Amsterdam, Amsterdam, The Netherlands
[17] Department of Microbiology, The Ohio State University, Columbus, OH 43210, USA
[18] Institute for Landscape Ecology and Resources Management (ILR), Research Centre for BioSystems, Land Use and Nutrition (iFZ), Justus Liebig University Giessen, Heinrich-Buff-Ring 26, 35392 Giessen, Germany
[19] Centre for International Development and Environmental Research (ZEU), Justus Liebig University Giessen, Senckenbergstrasse 3, 35390 Giessen, Germany
[20] Federal Agency for Water Management, Institute for Land and Water Management Research, Petzenkirchen, 3252, Austria
[21] Faculty of Agricultural and Environmental Sciences, University of Rostock, Justus-von-

Liebig-Weg 6, 18059 Rostock, Germany
[22] Climate Resilience, Wageningen Environmental Research, Wageningen University & Research, PO Box 47, NL-6700 AA, Wageningen, the Netherland
[23] Stiftung Naturschutz Schleswig-Holstein, Eschenbrook 4, 24113 Molfsee, Germany
[24] Centro Austral de Investigaciones Científicas (CADIC), Consejo Nacional de Investigaciones Científicas y Técnicas (CONICET), Ushuaia, Tierra del Fuego, Argentina
[25] Instituto de Ciencias Polares, Ambiente y Recursos Naturales (ICPA), Universidad Nacional de Tierra del Fuego (UNTDF), Ushuaia, Tierra del Fuego, Argentina
[26] Départment de géographie, Université de Montréal, Canada
[27] Department of Ecohydrology and Biogeochemistry, Leibniz Institute of Freshwater Ecology and Inland Fisheries, Berlin, Germany
[28] Department of Ecology, Radboud Institute for Biological and Environmental Sciences, Faculty of Science, Radboud University Nijmegen, 6525 AJ Nijmegen, The Netherlands
[29] School of Biological Sciences, Faculty of Environmental and Life Sciences, University of Southampton, Southampton, SO17 1BJ, UK
[30] Département de Géographie, Université de Montréal, Campus MIL, 1375 Avenue Thérèse Lavoie-Roux, Montréal, Québec, H2V 0B3, Canada
[31] University of Alberta, Department of Renewable Resources, South Academic Building 348D, Edmonton, AB, T6G 2G7
[32] Hatfield Consultants, 1228 Kensington Rd NW Unit 305, Calgary, AB T2N 3P7
[33] Department of Earth Sciences, University of Durham, Durham, DH1 3LE, UK
[34] Ecohydrology Research Group, Dept. Hydrology & Water Resources, Nanjing University of Information Science and Technology, Ningliu Road. 219, 210000 Nanjing
* corresponding author(s): Henning Teickner (henning.teickner@uni-muenster.de)

Attributes in the 'pmird' database

Table S1: Definition of all attributes in the 'pmird' database.

Attribute name	Description	Unit
abbreviation	In table 'custom_units': A string representing an abbreviation for the custom unit.	-
abstract	A free text field with an abstract for the dataset.	-
acknowledgements	A free text field with acknowledgements for a dataset.	-
activity_137Cs	A numeric value representing the measured mean value of the ^{137}Cs activity of the sample [DPM g^{-1}] (DPM are disintegrations per minute).	g^{-1} min^{-1}
activity_-137Cs_err	A numeric value representing the measurement error of the ^{137}Cs activity of the sample [DPM g^{-1}] (DPM are disintegrations per minute).	g^{-1} min^{-1}

(Continued on Next Page...)

Table S1: *(continued)*

Attribute name	Description	Unit
activity_210Pb	A numeric value representing the measured mean value of the ^{210}Pb activity of the sample [Bq kg^{-1}].	Bq kg^{-1}
activity_-210Pb_err	A numeric value representing the measurement error of the ^{210}Pb activity of the sample [Bq kg^{-1}].	Bq kg^{-1}
activity_226Ra	A numeric value representing the measured mean value of the ^{226}Ra activity of the sample [DPM g^{-1}] (DPM are disintegrations per minute).	g^{-1} min^{-1}
activity_-226Ra_err	A numeric value representing the measurement error of the ^{226}Ra activity of the sample [DPM g^{-1}] (DPM are disintegrations per minute).	g^{-1} min^{-1}
Ag	A numeric value representing the silver mass content of the sample [mass-ppm].	μg g^{-1}
Ag_err	A numeric value representing the measurement error of the silver mass content value of the sample [mass-ppm].	μg g^{-1}
age_14C	A numeric value representing the mean value of the uncalibrated ^{14}C age of the sample [yr BP].	yr
age_14C_err	A numeric value representing the error of the uncalibrated ^{14}C age of the sample [yr].	yr
Al	A numeric value representing the aluminium mass content of the sample [mass-ppm].	μg g^{-1}
Al_err	A numeric value representing the measurement error of the aluminium mass content value of the sample [mass-ppm].	μg g^{-1}
apodisation_-function	A string representing the name of the apodisation function.	-
As	A numeric value representing the arsenic mass content of the sample [mass-ppm].	μg g^{-1}
As_err	A numeric value representing the measurement error of the arsenic mass content value of the sample [mass-ppm].	μg g^{-1}
attribute_-definition	A free text field with a textual description of the meaning of attributes in the pmird database.	-
attribute_name	A string describing the names of the attributes in all tables of the pmird database.	-
Ba	A numeric value representing the barium mass content of the sample [mass-ppm].	μg g^{-1}
Ba_err	A numeric value representing the measurement error of the barium mass content value of the sample [mass-ppm].	μg g^{-1}

(Continued on Next Page...)

Table S1: *(continued)*

Attribute name	Description	Unit
background_-activity_-reached_210Pb	A logical value (TRUE or FALSE) indicating if the corresponding ^{210}Pb activity in the same row (activity_210Pb) represents (according to the interpretation of the person who dated the core) the supported (background) ^{210}Pb activity (TRUE) or not (FALSE).	-
beamsplitter_-name	A string representing the name of the beamsplitter.	-
begin_date	The minimum value of "sampling_date" in table "samples" for a specific dataset.	-
bibtex	A string representing the bibtex code used for a literature reference throughout the pmird database.	-
bounds_-maximum	A numeric value representing the minimum possible value for a numeric attribute.	-
bounds_-minimum	A numeric value representing the maximum possible value for a numeric attribute.	-
Br	A numeric value representing the bromine mass content of the sample [mass-ppm].	μg g^{-1}
Br_err	A numeric value representing the measurement error of the bromine mass content value of the sample [mass-ppm].	μg g^{-1}
bulk_density	A numeric value representing the bulk density of the sample [g cm^{-3}].	g/cm^3
bulk_density_-210Pb	A numeric value representing the mass density of the subsample on which the ^{210}Pb activity of the sample was measured [g cm^{-3}].	g/cm^3
bulk_density_-210Pb_err	A numeric value representing the error of the mass density of the subsample on which the ^{210}Pb activity of the sample was measured [g cm^{-3}].	g/cm^3
bulk_density_-err	A numeric value representing the measurement error of the bulk density of the sample [g cm^{-3}].	g/cm^3
C	A numeric value representing the carbon mass content of the sample [mass-%].	g g^{-1}
C_err	A numeric value representing the measurement error of the carbon mass content value of the sample [mass-%].	g g^{-1}
Ca	A numeric value representing the calcium mass content of the sample [mass-ppm].	μg g^{-1}

(Continued on Next Page...)

Table S1: *(continued)*

Attribute name	Description	Unit
Ca_err	A numeric value representing the measurement error of the calcium mass content value of the sample [mass-ppm].	$\mu g\ g^{-1}$
CaCO3	A numeric value representing the $CaCO_3$ mass content of the sample [µg g^{-1}]	$\mu g\ g^{-1}$
CaCO3_err	A numeric value representing the measurement error of the $CaCO_3$ mass content value of the sample [mass-ppm].	$\mu g\ g^{-1}$
change_date	A string with the date when a dataset was changed with the format YYYY-MM-DD.	-
change_scope	In table 'maintenances': A string describing the scope to which the documented change was applied.	-
Cl	A numeric value representing the chlorine mass content of the sample [mass-ppm].	$\mu g\ g^{-1}$
Cl_err	A numeric value representing the measurement error of the chlorine mass content value of the sample [mass-ppm].	$\mu g\ g^{-1}$
comment	In table 'change_histories': A string with any comments on changes made to a dataset.	-
comments_-measurements	A free text field where you can enter all information related to the sample that is not covered by the remaining fields. For example you could provide information on potential contamination sources, issues with specific parameters, additional information to the sampling site, e.g. present vegetation, past vegetation, specific conditions during sampling, ...	-
comments_-samples	A free text field where you can enter all information related to the sample that is not covered by the remaining fields. For example you could provide information on potential contamination sources, issues with specific parameters, additional information to the sampling site, e.g. present vegetation, past vegetation, specific conditions during sampling, ...	-
common_name	A string representing a common taxon name.	-
core_label	A string representing a label for the peat core (if the sample was taken from a peat core). This can be a custom label.	-
counting_-method_210_-Pb	A string representing a description of the counting method used for measuring the ^{210}Pb activities (one of alpha, beta or gamma).	-

(Continued on Next Page...)

Table S1: *(continued)*

Attribute name	Description	Unit
coverage_type	A string describing the type of a specific coverage (an entry in one of the tables "geographic_coverage", "temporal_coverage", or "taxonomic_coverage"). Must be one of "geographic_coverage", "temporal_coverage", "taxonomic_coverage".	-
Cr	A numeric value representing the chromium mass content of the sample [mass-ppm].	$\mu g\ g^{-1}$
Cr_err	A numeric value representing the measurement error of the chromium mass content value of the sample [mass-ppm].	$\mu g\ g^{-1}$
Cu	A numeric value representing the copper mass content of the sample [mass-ppm].	$\mu g\ g^{-1}$
Cu_err	A numeric value representing the measurement error of the copper mass content value of the sample [mass-ppm].	$\mu g\ g^{-1}$
d13C	A numeric value representing the ^{13}C isotope signature of the sample in delta permil relative to the VPDB standard.	dimensionless
d13C_err	A numeric value representing the measurement error of the ^{13}C isotope signature of the sample in delta permil.	dimensionless
d15N	A numeric value representing the ^{15}N isotope signature of the sample in delta permil relative to air.	dimensionless
d15N_err	A numeric value representing the measurement error of the ^{15}N isotope signature of the sample in delta permil.	dimensionless
d18O	A numeric value representing the ^{18}O isotope signature of the sample in delta permil relative to VSMOW.	dimensionless
d18O_err	A numeric value representing the measurement error of the ^{18}O isotope signature of the sample in delta permil.	dimensionless
d2H	A numeric value representing the ^{2}H isotope signature of the sample in delta permil relative to VSMOW.	dimensionless
d2H_err	A numeric value representing the measurement error of the ^{2}H isotope signature of the sample in delta permil.	dimensionless
data_point_-number	A numeric value representing the number of data points in the spectrum.	-
description	A free text field. In table "custom_units": A description of a custom unit. In table "maintenances": A description of the maintenance of a dataset. In table "method_steps": A description of a method step. In table "quality_control": A description of the quality control.	-

(Continued on Next Page...)

Table S1: *(continued)*

Attribute name	Description	Unit
detector_-gain_factor	A numeric value representing the detection gain factor.	dimensionless
detector_name	A string representing the name of the detector.	-
dimension	A string representing the dimension of the unit.	-
east_-bounding_-coordinate	A numeric value representing the east bounding values of a bonuding box around the sampling locations for a dataset (in the EPSG:3857 projection coordinate system — this is the system used by Google and is based on the WGS 84 reference system) [°E]. This is the minimum value in "sampling_longitude" from table "samples" for s specific dataset.	-
electron_-accepting_-capacity	A numeric value representing the electron accepting capacity (EAC) of the sample [μmol (g C)$^{-1}$].	μmol g^{-1}
electron_-accepting_-capacity_err	A numeric value representing the measurement error of the electron accepting capacity (EAC) of the sample [μmol (g C)$^{-1}$].	μmol g^{-1}
electron_-donating_-capacity	A numeric value representing the electron donating capacity (EDC) of the sample [μmol (g C)$^{-1}$].	μmol g^{-1}
electron_-donating_-capacity_err	A numeric value representing the measurement error of the electron donating capacity (EDC) of the sample [μmol (g C)$^{-1}$].	μmol g^{-1}
electronic_-mail_address	A string representing the email address for a person.	-
end_date	The maximum value of "sampling_date" in table "samples" for a specific dataset.	-
enthalpy_of_-formation	A numeric value representing the standard enthalpy of formation of the sample (using a molecular formular informed by element measurements of the sample) [kJ mol^{-1}].	kJ mol^{-1}
enthalpy_of_-formation_err	A numeric value representing the measurement error of the standard enthalpy of formation of the sample (using a molecular formular informed by element measurements of the sample) [kJ mol^{-1}].	kJ mol^{-1}
entropy_of_-formation	A numeric value representing the standard entropy of formation of the sample [J K^{-1} mol^{-1}].	J K^{-1} mol^{-1}
entropy_of_-formation_err	A numeric value representing the standard entropy of formation of the sample [J K^{-1} mol^{-1}].	J K^{-1} mol^{-1}

(Continued on Next Page...)

Table S1: *(continued)*

Attribute name	Description	Unit
explanation	In table 'missing_value_codes': A string explaining what the corresponding missing value code means.	-
exponentiation_-factor	A numeric value representing the exponentiation factor used for file compression.	-
Fe	A numeric value representing the iron mass content of the sample [mass-ppm].	μg g^{-1}
Fe_err	A numeric value representing the measurement error of the iron mass content value of the sample [mass-ppm].	μg g^{-1}
Fe2	A numeric value representing the Fe^{2+} mass content in the sample [mass-ppm]	μg g^{-1}
Fe2_err	A numeric value representing the measurement error of the Fe^{2+} mass content value of the sample [mass-ppm].	μg g^{-1}
Fe3	A numeric value representing the Fe^{3+} mass content in the sample [mass-ppm]	μg g^{-1}
Fe3_err	A numeric value representing the measurement error of the Fe^{3+} mass content value of the sample [mass-ppm].	μg g^{-1}
format_string	A string defining the format of a nominal variable.	-
general_-taxonomic_-coverage	In table 'taxonomic_coverage': A string describing the range of taxa addressed in the data set or collection.	-
geographic_-description	A free text field where the gepgraphic coverage for a dataset is described.	-
getting_started	A free text field where instructions to use the data are described.	-
Gibbs_-energy_of_-formation	A numeric value representing the standard Gibbs energy of formation of the sample [kJ mol^{-1}].	kJ mol^{-1}
Gibbs_-energy_of_-formation_err	A numeric value representing the measurement error of the standard Gibbs energy of formation of the sample [kJ mol^{-1}].	kJ mol^{-1}
given_name	A string rerpesenting the given name(s) of a person.	-
H	A numeric value representing the hydrogen mass content of the sample [mass-%].	g g^{-1}
H_err	A numeric value representing the measurement error of the hydrogen mass content value of the sample [mass-%].	g g^{-1}
heat_of_-combustion	A numeric value representing the heat of combustion of the sample (using a molecular formular informed by element measurements of the sample) [kJ mol^{-1}].	kJ mol^{-1}

(Continued on Next Page...)

Table S1: *(continued)*

Attribute name	Description	Unit
heat_of_-combustion_err	A numeric value representing the measurement error of the heat of combustion of the sample (using a molecular formular informed by element measurements of the sample) [kJ mol^{-1}].	kJ mol^{-1}
Hg	A numeric value representing the mercury mass content of the sample [mass-ppm].	μg g^{-1}
Hg_err	A numeric value representing the measurement error of the mercury mass content value of the sample [mass-ppm].	μg g^{-1}
holocellulose_-content	A numeric value representing the holocellulose content of the sample [mass-%].	g g^{-1}
holocellulose_-content_err	A numeric value representing measurement error of the holocellulose content of the sample [mass-%].	g g^{-1}
humidity_-absolute_-reference	A numeric value representing the absolute humidity during measurement of the reference (background).	g L^{-1}
humidity_-absolute_-sample	A numeric value representing the absolute humidity during measurement of the sample.	g L^{-1}
humidity_-relative_-reference	A numeric value representing the relative humidity during measurement of the reference (background).	kPa kPa^{-1}
humidity_-relative_sample	A numeric value representing the relative humidity during measurement of the sample.	kPa kPa^{-1}
hydraulic_-conductivity	A numeric value representing the saturated hydraulic conductivity (K_s) of the sample [cm h^{-1}].	cm h^{-1}
hydraulic_-conductivity_-err	A numeric value representing the error of the saturated hydraulic conductivity (K_s) of the sample [cm h^{-1}].	cm h^{-1}
id_attribute	An integer value representing an id for each attribute in the pmird database.	-
id_change_-history	An integer value representing an id for each entry in the table “change_histories“ in the pmird database.	-
id_citation	An integer value representing an id for each entry in the table “citations“ in the pmird database.	-
id_coverage	An integer value representing an id for each entry in the table “coverages“ in the pmird database.	-

(Continued on Next Page...)

Table S1: *(continued)*

Attribute name	Description	Unit
id_dataset	A numeric id for the dataset (starting with 1 and increasing by 1; for one data contribution, this should be 1 for all samples and the appropriate id is assigned when the data are merged into the database).	-
id_-geographic_-coverage	An integer value representing an id for each entry in the table “geographic_coverages“ in the pmird database.	-
id_instrument	An integer value representing an id for each entry in the table “instruments“ in the pmird database.	-
id_license	An integer value representing an id for each entry in the table “licenses“ in the pmird database.	-
id_-macrofossil_-type	A numeric id for the macrofossil type (starting with 1).	-
id_-maintenance	An integer value representing an id for each entry in the table “maintenances“ in the pmird database.	-
id_-measurement	A numeric id for measurements (starting with 1 and increasing by 1). This means that each measurement gets its own rows and measurements for different attributes are considered independent, i.e. multiple measurement ids for the same sample just count replicate measurements for any attribute. For attributes with less measurements than for a different attribute, just fill measurements starting from smaller id_measurement and leave the cells in the remaining rows empty.	-
id_-measurement_-scale	An integer value representing an id for each entry in the table “measurement_scales“ in the pmird database.	-
id_method	An integer value representing an id for each entry in the table “methods“ in the pmird database.	-
id_method_-step	An integer value representing an id for each entry in the table “mehod_steps“ in the pmird database.	-
id_missing_-value_code	An integer value representing an id for each entry in the table “missing_value_codes“ in the pmird database.	-
id_person	An integer value representing an id for each entry in the table “persons“ in the pmird database.	-
id_quality_-control	An integer value representing an id for each entry in the table “quality_controls“ in the pmird database.	-

(Continued on Next Page...)

Table S1: *(continued)*

Attribute name	Description	Unit
id_sample	A numeric id for the sample (starting with 1 and increasing by 1).	-
id_site	A numeric id for the site where the sample was collected. This should be the corresponding value in the file site_info.csv.	-
id_-taxonomic_-classification	An integer value representing an id for each entry in the table "taxonomic_classifications" in the pmird database.	-
id_-taxonomic_-coverage	An integer value representing an id for each entry in the table "taxonomic_coverages" in the pmird database.	-
id_temporal_-coverage	An integer value representing an id for each entry in the table "temporal_coverages" in the pmird database.	-
id_unit	An integer value representing an id for each entry in the table "units" in the pmird database.	-
identifier	In table 'licenses': A string representing an identifier for the license.	-
In	A numeric value representing the indium mass content of the sample [mass-ppm].	μg g^{-1}
In_err	A numeric value representing the measurement error of the indium mass content value of the sample [mass-ppm].	μg g^{-1}
instrumentation	A string describing an instrument in the table "instruments" in the pmird database.	-
intellectual_-rights	A free text field describing any intellectual rights connected to a dataset.	-
introduction	A free text field providing an intridunctory description to a dataset.	-
is_baseline_-corrected	A logical value indicating if a spectrum is already baseline corrected (TRUE) or not (FALSE).	-
K	A numeric value representing the potassium mass content of the sample [mass-ppm].	μg g^{-1}
K_err	A numeric value representing the measurement error of the potassium mass content value of the sample [mass-ppm].	μg g^{-1}
Klason_-lignin_content	A numeric value representing the Klason lignin content of the sample [mass-%].	g g^{-1}

(Continued on Next Page...)

Table S1: *(continued)*

Attribute name	Description	Unit
Klason_-lignin_-content_err	A numeric value representing measurement error of the Klason lignin content of the sample [mass-%].	g g^{-1}
lab_code_14C	A string representing a code for the laboratory where the ^{14}C activities were measured (^{14}C ages were determined).	-
laboratory_-label_210Pb	A string representing a label (preferentially a code, similarly to lab_code_14C) for the laboratory where the ^{210}Pb activities were measured.	-
language	A string representing a description for the language used in a dataset.	-
laser_-wavenumber	A numeric value representing the wavenumber of the laser.	-
license_name	A string representing the name of a license.	-
loss_on_-ignition	A numeric value representing the loss on ignition of the sample [mass-%].	g g^{-1}
loss_on_-ignition_err	A numeric value representing the measurement error of the loss on ignition value of the sample [mass-%].	g g^{-1}
macrofossil_-count	A numeric value representing a count for a specific macrofossil type (e.g. Carex seeds) divided by the volume of peat for which the macrofossil type was counted [cm^{-13}].	-
macrofossil_-presence	A logical value indicating if the respective macrofossil type was present in the peat sample (TRUE) or not (FALSE).	-
macrofossil_-size_lower	A numeric value representing the lower size bound for the macrofossil type (e.g. if only macrocharcoal particles larger than 1mm were considered) [mm].	mm
macrofossil_-size_upper	A numeric value representing the upper size bound for the macrofossil type (e.g. if wood fragments were classified by size) [mm].	mm
macrofossil_-taxon_organ	The same as attribute 'taxon_organ', but for macrofossils.	-
macrofossil_-type	A string representing the macrofossil type. This can be a custom value, such as 'vegetation', 'ash', 'charcoal', 'unidentifiable organic matter'.	-
macrofossil_-volume_-fraction	A numeric value representing the volumetric fraction of a specific macrofossil type in the peat sample [L L^{-1}]	L L^{-1}

(Continued on Next Page...)

Table S1: *(continued)*

Attribute name	Description	Unit
macrofossil_-volume_-fraction_err	A numeric value representing measurement error for the volumetric fraction of a specific macrofossil type in the peat sample [L L^{-1}]	L L^{-1}
macroporosity	A numeric value representing the macroporosity of the sample [volume-%]. Since the term 'macroporosity' is ambiguous, the term should be defined in the description of the methods if values are available.	cm^3 cm^{-3}
macroporosity_-err	A numeric value representing the error of the macroporosityporosity of the sample [volume-%].	cm^3 cm^{-3}
maintenance_-update_-frequency	In table 'maintenances': A string describing the frequency with which changes and additions are made to the dataset after the initial dataset is completed.	-
mass	A numeric value representing the mass of the sample [g]. This is the mass of the extracted sample and masses of subsets of the sample may differ (for example: A peat layer dried at 105°C may have a mass of 200 g. This is the value to be reported. After milling, 10 g may be used to measure the pH value (this value should not be reported here, but in the description of the methods or in column 'comments'.))	g
mass_210Pb	A numeric value representing the measured mass of the subsample on which the ^{210}Pb activity of the sample was measured [g].	g
mass_210Pb_-err	A numeric value representing the measurement error of the mass of the subsample on which the ^{210}Pb activity of the sample was measured [g].	g
mass_err	A numeric value representing the measurement error of the mass of the sample [g].	g
measurement_-date	A datetime representing the dat and time when the spectrum was measured.	-
measurement_-date_reference	A datetime representing the dat and time when the reference (background) spectrum was measured when a separate background spectrum is available in the file.	-
measurement_-device	A string representing the name of the measurement device.	-
measurement_-scale	In table 'measurement_scales': A string representing the measurmeent scale for a value.	-
Mg	A numeric value representing the magnesium mass content of the sample [mass-ppm].	μg g^{-1}

(Continued on Next Page...)

Table S1: *(continued)*

Attribute name	Description	Unit
Mg_err	A numeric value representing the measurement error of the magnesium mass content value of the sample [mass-ppm].	μg g^{-1}
mir_co2_-contribution_-relative	A numeric value representing the relative carbon dioxide contribution to a mid infrared spectrum. This is the slope of an ordinary least squares regression model fitting a reference carbon dioxide spectrum to a specified range of the spectrum.	-
mir_mode	A string representing the measurement mode in which the mid infrared spectra were measured. One of "absorbance_ftir", "atr_ftir", "dr_ftir".	-
mir_water_-vapor_-contribution_-relative	A numeric value representing the relative water vapor contribution to a mid infrared spectrum. This is the slope of an ordinary least squares regression model fitting a reference water vapor spectrum to a specified range of the spectrum.	-
mir_water_-vapor_-contribution_-relative_sd	A numeric value representing an uncertainty estimate for the relative water vapor contribution to a mid infrared spectrum. This is the standard deviation of the slope of an ordinary least squares regression model fitting a reference water vapor spectrum to a specified range of the spectrum.	-
mir_water_-vapor_-contribution_-relative_sd	A numeric value representing an uncertainty estimate for the relative water vapor contribution to a mid infrared spectrum. This is the standard deviation of the slope of an ordinary least squares regression model fitting a reference water vapor spectrum to a specified range of the spectrum.	-
mirs_file	A string representing the path to the file with the mid infrared spectrum for the sample. This is a relative path relative to the root of the database. This field should be left empty upon creating the project because the file path is added automatically when the data are included in the database.	-
missing_-value_code	A string representing a code for missing value in the tables "data", "samples", "macrofossils", and "mir_metadata" in the pmird database.	-
Mn	A numeric value representing the manganese mass content of the sample [mass-ppm].	μg g^{-1}

(Continued on Next Page...)

Table S1: *(continued)*

Attribute name	Description	Unit
Mn_err	A numeric value representing the measurement error of the manganese mass content value of the sample [mass-ppm].	μg g^{-1}
multiplier_to_-si	A numeric value representing the value with which a given value with a certain measurement unit has to be multiplied in order to convert it to a related SI unit.	dimensionless
N	A numeric value representing the nitrogen mass content of the sample [mass-%].	g g^{-1}
N_err	A numeric value representing the measurement error of the nitrogen mass content value of the sample [mass-%].	g g^{-1}
Na	A numeric value representing the sodium mass content of the sample [mass-ppm].	μg g^{-1}
Na_err	A numeric value representing the measurement error of the sodium mass content value of the sample [mass-ppm].	μg g^{-1}
Ni	A numeric value representing the nickel mass content of the sample [mass-ppm].	μg g^{-1}
Ni_err	A numeric value representing the measurement error of the nickel mass content value of the sample [mass-ppm].	μg g^{-1}
noise_level_-relative	A numeric value representing the relative noise level of a mid infrared spectrum.	-
north_-bounding_-coordinate	A numeric value representing the north bounding values of a bonuding box around the sampling locations for a dataset (in the EPSG:3857 projection coordinate system — this is the system used by Google and is based on the WGS 84 reference system) [°N]. This is the maximum value in “sampling_latitude” from table ”samples” for s specific dataset.	-
number_type	A string representing the number type of a numeric variable.	-
O	A numeric value representing the oxygen mass content of the sample [mass-%].	g g^{-1}
O_err	A numeric value representing the measurement error of the oxygen mass content value of the sample [mass-%].	g g^{-1}
old_value	In table ‘change_histories‘: A string describing the old dataset before the change in the current version.	-
online_url	In table ‘persons‘: A link to the website of a person.	-
P	A numeric value representing the phosphorous mass content of the sample [mass-ppm].	μg g^{-1}

(Continued on Next Page...)

Table S1: *(continued)*

Attribute name	Description	Unit
P_err	A numeric value representing the measurement error of the phosphorous mass content value of the sample [mass-ppm].	μg g^{-1}
parent_si	A string representing the SI unit from which a certain derived unit is derived.	-
Pb	A numeric value representing the lead mass content of the sample [mass-ppm].	μg g^{-1}
Pb_err	A numeric value representing the measurement error of the lead mass content value of the sample [mass-ppm].	μg g^{-1}
pH	A numeric value representing the pH value of the sample.	dimensionless
pH_err	A numeric value representing the measurement error of the pH value value of the sample.	dimensionless
phone	A string representing the phone number of a person.	-
porosity	A numeric value representing the porosity of the sample [volume-%].	cm^3 cm^{-3}
porosity_err	A numeric value representing the error of the porosity of the sample [volume-%].	cm^3 cm^{-3}
power	In table ‘unit_types‘: An integer value. The power to which a dimension is raised.	-
pub_date	A string with the year when the dataset was originally published with the format YYYY.	-
purge_delay	A numeric value representing the duration of purge delay before a measurement in seconds.	s
purpose	A free text field dscribing the purpse for which the dataset was created.	-
Rb	A numeric value representing the rubidium mass content of the sample [mass-ppm].	μg g^{-1}
Rb_err	A numeric value representing the measurement error of the rubidium mass content value of the sample [mass-ppm].	μg g^{-1}
reference_-publication	A string in the bibtex format giving informatio on references which serve as references for data or publication where a certain dataset is described or used.	-
rep_no	An integer value representing the sample repetition number.	-
S	A numeric value representing the sulfur mass content of the sample [mass-ppm].	μg g^{-1}

(Continued on Next Page...)

Table S1: *(continued)*

Attribute name	Description	Unit
S_err	A numeric value representing the measurement error of the sulfur mass content value of the sample [mass-ppm].	μg g^{-1}
salutation	A string representing the salutation used to address an individual.	-
sample_-depth_lower	A numeric value representing the depth of the lower boundary of a sample relative to the land surface (e.g. peat surface) [cm].	cm
sample_-depth_lower_-err	A numeric value representing the measurement error of the lower boundary of a sample [cm].	cm
sample_-depth_upper	A numeric value representing the depth of the upper boundary of a sample relative to the land surface (e.g. peat surface) [cm].	cm
sample_-depth_upper_-err	A numeric value representing the measurement error of the upper boundary of a sample [cm].	cm
sample_label	A string representing a label for each sample.	-
sample_-microhabitat	A string describing the microhabitat where the sample was collected. For peat, this should be one of 'hummock', 'hollow', 'lawn', 'pond'. In other cases, a custom value can be used.	-
sample_-treatment	A string with an description of an experimental tratment if this was applied. By default, this should be 'control', indicating that there was no manipulation. If there was any experimental manipulation, this can be abbreviated by a label (e.g. by a treatment level) that is defined in the textual description of the project (in the file 'description.docx').	-
sample_type	A string describing the type of the sample. Must be one of 'peat', 'dom', 'vegetation', 'litter'.	-
sample_type2	A string describing the type of the sample. Here you can provide individual (own) categories which may provide more details than the column sample_type.	-
sampling_-altitude	A numeric value representing the altitude of the exact sampling position [m above sea level].	m
sampling_-altitude_err	A numeric value representing the measurement error of the altitude of the exact sampling position [m above sea level].	m

(Continued on Next Page...)

Table S1: *(continued)*

Attribute name	Description	Unit
sampling_date	A string with the date when the sample was collected (in the field) with the format YYYY-MM-DD.	-
sampling_-description	A free text field where the collection of samples is described (including experimental or sampling design).	-
sampling_-latitude	A numeric value representing the latitude coordinates of the exact sampling position (in the EPSG:3857 projection coordinate system — this is the system used by Google and is based on the WGS 84 reference system) [°N].	-
sampling_-latitude_err	A numeric value representing the measurement error of the latitude coordinates of the exact sampling position (in the EPSG:3857 projection coordinate system — this is the system used by Google and is based on the WGS 84 reference system) [°N].	-
sampling_-longitude	A numeric value representing the longitude coordinates of the exact sampling position (in the EPSG:3857 projection coordinate system — this is the system used by Google and is based on the WGS 84 reference system) [°W].	-
sampling_-longitude_err	A numeric value representing the measurement error of the longitude coordinates of the exact sampling position (in the EPSG:3857 projection coordinate system — this is the system used by Google and is based on the WGS 84 reference system) [°W].	-
Sb	A numeric value representing the antimony mass content of the sample [mass-ppm].	μg g^{-1}
Sb_err	A numeric value representing the measurement error of the antimony mass content value of the sample [mass-ppm].	μg g^{-1}
scan_number	An integer value representing the number of scans.	-
scan_speed	A numeric value representing the scan speed.	kHz
Si	A numeric value representing the silicium mass content of the sample [mass-ppm].	μg g^{-1}
Si_err	A numeric value representing the measurement error of the silicium mass content value of the sample [mass-ppm].	μg g^{-1}
Sn	A numeric value representing the tin mass content of the sample [mass-ppm].	μg g^{-1}

(Continued on Next Page...)

Table S1: *(continued)*

Attribute name	Description	Unit
Sn_err	A numeric value representing the measurement error of the tin mass content value of the sample [mass-ppm].	μg g^{-1}
source_name	A string representing the name of the infrared radiation source.	-
south_-bounding_-coordinate	A numeric value representing the south bounding values of a bonuding box around the sampling locations for a dataset (in the EPSG:3857 projection coordinate system — this is the system used by Google and is based on the WGS 84 reference system) [°N]. This is the minimum value in “sampling_latitude” from table ”samples” for s specific dataset.	-
Sr	A numeric value representing the strontium mass content of the sample [mass-ppm].	μg g^{-1}
Sr_err	A numeric value representing the measurement error of the strontium mass content value of the sample [mass-ppm].	μg g^{-1}
standard_unit	A logical value indicating whether the unit is a standard unit of the Ecological Metadata Language or not.	-
sur_name	A string representing the sur name of a person.	-
taxon_organ	A string describing the organ of a taxon the sample represents (if the sample represents a taxon). For example, if the sample is Carex lasiocarpa, this could be ’shoot’, or ’root’, or ’leaves’.	-
taxon_rank_-name	A string describing the taxon rank the value in column taxon_rank_value represents (if the sample can be assigned to a specific taxon). For exampe, if the value in column taxon_rank_value is a species name, then you should enter ’species’ here, or if the value in column taxon_rank_value is a genus name, then you should enter ’genus’ here.	-
taxon_rank_-value	A string describing the taxon rank value of the sample (if the sample can be assigned to a taxon). For example, if the sample is a distinct species, enter the scientific species name here, or if the sample can be assigned to a genus, enter the scientific genus name here.	-
temperature_-scanner_-reference	A numeric value representing the temperature of the scanner during the measurement of the reference (background).	K

(Continued on Next Page...)

Table S1: *(continued)*

Attribute name	Description	Unit
temperature_-scanner_-sample	A numeric value representing the temperature of the scanner during the measurement of the sample.	K
text_domain_-definition	A string representing the text domain for a string.	-
Ti	A numeric value representing the titanium mass content of the sample [mass-ppm].	μg g^{-1}
Ti_err	A numeric value representing the measurement error of the titanium mass content value of the sample [mass-ppm].	μg g^{-1}
title	A free text field with the title for a dataset.	-
udunits_unit	A string representing a measurement unit in the udunits format.	-
unit_type	A string representing the type of a unit.	-
url	In table ‘licenses‘: A string representing an url to a website with information on the license.	-
user_id	In table ‘persons‘: A string representing an identifier that links this party to a directory of individuals.	-
volume	A numeric value representing the volume of the sample [cm^3].	cm^3
volume_210Pb	A numeric value representing the measured volume of the subsample on which the ^{210}Pb activity of the sample was measured [cm^3].	cm^3
volume_-210Pb_err	A numeric value representing the error of the volume of the subsample on which the ^{210}Pb activity of the sample was measured [cm^3].	cm^3
volume_err	A numeric value representing the measurement error of the volume of the sample [cm^3].	cm^3
water_content	A numeric value representing the water mass content of the sample as mass of water divided by the mass of the wet sample [g g^{-1}]	g g^{-1}
water_-content_err	A numeric value representing the measurement error of the water mass content value of the sample [g g^{-1}].	g g^{-1}

(Continued on Next Page...)

Table S1: *(continued)*

Attribute name	Description	Unit
west_-bounding_-coordinate	A numeric value representing the west bounding values of a bonuding box around the sampling locations for a dataset (in the EPSG:3857 projection coordinate system — this is the system used by Google and is based on the WGS 84 reference system) [°E]. This is the maximum value in "sampling_longitude" from table "samples" for s specific dataset.	-
x_variable_-max	A numeric value representing the maximum x variable value of each spectrum.	cm^{-1}
x_variable_-min	A numeric value representing the minimum x variable value of each spectrum.	cm^{-1}
x_variable_-type	A string representing the type of the x variable.	-
y_variable_-type	A string representing the type of the y variable.	-
year_137Cs	A numeric value representing the age that was assigned to the sample based on the ^{137}Cs activity inventory [yr AD] (e.g. by relating it to the date of the Chernobyl accident).	yr
zero_filling_-factor	An integer value representing the zero filling factor.	-
Zn	A numeric value representing the zinc mass content of the sample [mass-ppm].	$\mu g\ g^{-1}$
Zn_err	A numeric value representing the measurement error of the zinc mass content value of the sample [mass-ppm].	$\mu g\ g^{-1}$
Zr	A numeric value representing the zirconium mass content of the sample [mass-ppm].	$\mu g\ g^{-1}$
Zr_err	A numeric value representing the measurement error of the zirconium mass content value of the sample [mass-ppm].	$\mu g\ g^{-1}$

Example of the method description for dataset 8

Table S2: Method and method steps description for dataset 8 in the 'pmird' database.

id_method	id_method_step	description
40	56	Two coring locations were chosen to contrast on the one hand former bog parts used for peat extraction, with 1 m old grown peat left, which are restored by ditch-blocking and flooding since the middle of the 1970's , and where only young birches grow (max. height 4 m) (coring location 1: cores 1 to 2), and on the other hand old peat bodies not used for peat extraction, but strongly drained and overgrown by taller trees with few actively growing *Sphagna* (coring location 2: cores 3 to 5). sampling_longitude and sampling_latitude values refer to the coring locations and the different peat cores were taken in a distance of 20 cm to this location. At each coring location, two to three cores were taken, where a peat core is a set of peat samples taken at the same position. At location 1, the upper 30 cm had a too small bulk density to be sampled with a peat corer. Instead, a grab sample with the help of a stainless steel knife was taken (core 1, segment 1). In the resulting hole, segment 2 of core 1 was taken with a Russian peat corer (50 cm length). 20 cm next to the location where core 1 was taken, a second core was taken with the Russian peat corer after removing the top 30 cm peat. Core 2 was used for measurement of element contents and isotope signatures, bulk density, water content, and mid infrare spectra. Core 1 was used exclusively for pH measurements, except for segment 1 which was also used for laboratory analyses.

(Continued on Next Page...)

Table S2: *(continued)*

id_method	id_method_step	description
		At location 2, a 50 cm peat core for pH measurements was taken with the Russian peat corer (core 3). A second core (core 4) consisting of 5 segments with a total length of 250 cm was taken at a distance of 20 cm. During this, the tip of the corer might have compressed subsequent peat layers. This peat core was cut into 2 cm sections (upper 50 cm) and 5 cm sections (below 50 cm). This core was used to measure element contents and isotope signatures, bulk density, water content, and mid infrare spectra. Due to low bulk densities of the uppermost layers of core 4 (consisting mainly of fallen leaves from trees and few *Sphagna*), the material would not have been sufficient for all laboratory analyses. Therefore 20 cm next to the coring location of core 4, we took a grab sample by cutting a 6 cm · 6 cm block of peat which was separated into 2 cm layers using a stainless steel knife (core 5).<br>Peat cores were transported horizontally, placed onto two boxes and cut by hand with a stainless steel knife using a folding rule as depth reference. Samples to be used for laboratory analyses were placed into Whirl-Paks which were placed into transportable cooling boxes. The samples were freezed after 6 (core 2, upper 30 cm of core 1) and 4 (core 4 and 5) hours.
40	1	Freeze drying
40	57	Freeze-dried samples were milled in a vibrating cup mill with tungsten carbide cups and balls.
40	58	Freeze-dried and milled samples were analyzed with an elemental analyzer coupled to an isotope ratio mass spectometer to measure C and C contents and stable isotope signatures against VPDB and air, respectively. For this, samples are catalytically combusted and contents are determined via gas chromatography. Isotope signatures and element contents were corrected with the R package elco Teickner and Knorr (2020) using standards measured during the same runs as the samples. Duplicate measurements are remeasurements due to signal loss during the measurements.

(Continued on Next Page...)

Table S2: *(continued)*

id_method	id_method_step	description
40	59	For each sample, 2 mg freeze-dried and milled sample material were mixed with 200 mg KBr (FTIR grade, Sigma Aldrich, St. Louis, MO, USA) using an agate mortar and pestle and pressed to a pellet. MIR spectra were measured with an FTIR spectrometer in absorbance mode by averaging 32 scans. The spectra were background corrected with a pure KBr pellet.
40	60	Water content was computed by dividing the difference between the wet and dry mass by the wet mass of each sample. Sample masses were measured with packaging (Whirl-Paks) and sample masses without packaging were computed by subtracting the mass of two empty, clean Whirl-Paks, each measured five times. Measurement errors were estimated for the Whirl-Pak masses as the sample standard deviation of the replicate measurements. The same errors were assumed for the other weighed masses. Errors were propagated during computations using the R package quantities.
40	61	Bulk densities were computed by dividing the dry mass by the volume for each sample. Sample masses were measured with packaging (Whirl-Paks) and sample masses without packaging were computed by subtracting the mass of two empty, clean Whirl-Paks, each measured five times. Measurement errors were estimated for the whirl pack masses as the sample standard deviation of the replicate measurements. The same errors were assumed for the other weighed masses. Errors were propagated during computations using the R package quantities. Volumes were computed from the dimensions of the samples in the field (not considering depth errors during peat core slicing or dimension errors for grab samples). Volume errors were not considered during error propagation.
40	62	pH values were measured in the field according to method 4C1a1a4 of the USDA Kellog Soil Survey Laboratory Methods Manual (version 5.0). For this, 2.5 mL wet sample material was mixed with 4 mL $0.015\,mol\,L^{-1}$ $CaCl_2$, stirred by hand, and left unmoved for one hour. After stirring again, the pH value was measured in the supernatant.
